# Lymphovascular and perineural invasion guides adjuvant therapy after neoadjuvant chemoimmunotherapy in esophageal squamous cell carcinoma

**DOI:** 10.1093/oncolo/oyag234

**Published:** 2026-06-13

**Authors:** Yizhou Huang, Maohui Chen, Zhenyuan Yang, Bingqiang Cai, Yongcong Zhang, Shuliang Zhang, Taidui Zeng, Jun Yu, Chun Chen, Bin Zheng

**Affiliations:** Department of Thoracic Surgery, Fujian Medical University Union Hospital, Fuzhou, 350001, China; Key Laboratory of Cardio-Thoracic Surgery (Fujian Medical University), Fujian Province University, Fuzhou, 350001, China; National Key Clinical Specialty of Thoracic Surgery, Fuzhou, 350001, China; Clinical Research Center, Thoracic Tumors of Fujian Province, Fuzhou, 350001, China; Department of Thoracic Surgery, Fujian Medical University Union Hospital, Fuzhou, 350001, China; Key Laboratory of Cardio-Thoracic Surgery (Fujian Medical University), Fujian Province University, Fuzhou, 350001, China; National Key Clinical Specialty of Thoracic Surgery, Fuzhou, 350001, China; Clinical Research Center, Thoracic Tumors of Fujian Province, Fuzhou, 350001, China; Department of Thoracic Surgery, Fujian Medical University Union Hospital, Fuzhou, 350001, China; Key Laboratory of Cardio-Thoracic Surgery (Fujian Medical University), Fujian Province University, Fuzhou, 350001, China; National Key Clinical Specialty of Thoracic Surgery, Fuzhou, 350001, China; Clinical Research Center, Thoracic Tumors of Fujian Province, Fuzhou, 350001, China; Department of Thoracic Surgery, The First Affiliated Hospital of Xiamen University, Xiamen, 361003, China; Department of Thoracic Surgery, Quanzhou First Hospital, Fujian, 362000, China; Department of Thoracic Surgery, Fujian Medical University Union Hospital, Fuzhou, 350001, China; Key Laboratory of Cardio-Thoracic Surgery (Fujian Medical University), Fujian Province University, Fuzhou, 350001, China; National Key Clinical Specialty of Thoracic Surgery, Fuzhou, 350001, China; Clinical Research Center, Thoracic Tumors of Fujian Province, Fuzhou, 350001, China; Department of Thoracic Surgery, Fujian Medical University Union Hospital, Fuzhou, 350001, China; Key Laboratory of Cardio-Thoracic Surgery (Fujian Medical University), Fujian Province University, Fuzhou, 350001, China; National Key Clinical Specialty of Thoracic Surgery, Fuzhou, 350001, China; Clinical Research Center, Thoracic Tumors of Fujian Province, Fuzhou, 350001, China; Department of Thoracic Surgery, Fujian Medical University Union Hospital, Fuzhou, 350001, China; Key Laboratory of Cardio-Thoracic Surgery (Fujian Medical University), Fujian Province University, Fuzhou, 350001, China; National Key Clinical Specialty of Thoracic Surgery, Fuzhou, 350001, China; Clinical Research Center, Thoracic Tumors of Fujian Province, Fuzhou, 350001, China; Department of Thoracic Surgery, Fujian Medical University Union Hospital, Fuzhou, 350001, China; Key Laboratory of Cardio-Thoracic Surgery (Fujian Medical University), Fujian Province University, Fuzhou, 350001, China; National Key Clinical Specialty of Thoracic Surgery, Fuzhou, 350001, China; Clinical Research Center, Thoracic Tumors of Fujian Province, Fuzhou, 350001, China; Department of Thoracic Surgery, Fujian Medical University Union Hospital, Fuzhou, 350001, China; Key Laboratory of Cardio-Thoracic Surgery (Fujian Medical University), Fujian Province University, Fuzhou, 350001, China; National Key Clinical Specialty of Thoracic Surgery, Fuzhou, 350001, China; Clinical Research Center, Thoracic Tumors of Fujian Province, Fuzhou, 350001, China

**Keywords:** esophageal squamous cell carcinoma, neoadjuvant chemoimmunotherapy, lymphovascular invasion, perineural invasion, adjuvant therapy

## Abstract

**Background:**

Lymphovascular (LVI) and perineural invasion (PNI) are adverse pathological features in esophageal squamous cell carcinoma (ESCC). We evaluated the prognostic significance of combined LVI/PNI (LNI) after neoadjuvant chemoimmunotherapy (nCIT) and the potential benefit of adjuvant therapy.

**Methods:**

We retrospectively analyzed 473 ESCC patients undergoing nCIT followed by curative resection. LNI status was assessed in resected specimens. Survival outcomes were compared between LNI-positive and -negative patients using propensity score matching and multivariable Cox models. External validation was performed using an independent patient-level dataset.

**Results:**

LNI was present in 21% of patients. LNI-positive patients had worse 3-year overall survival (OS) (49.8% vs 76.4%) and disease-free survival (DFS) (41.7% vs 69.3%), which remained significant after matching. LNI independently predicted inferior OS (hazard ratio [HR] 1.52, *P* = .022) and DFS (HR 1.40, *P* = .045). Recurrence occurred more frequently in LNI-positive patients (39.4% vs 19.8%, *P* < .001), predominantly locoregional. Among LNI-positive patients, adjuvant therapy improved 3-year OS (56.8% vs 32.4%) and DFS (49.7% vs 20.7%; adjusted HR 0.43, *P* = .002). External validation confirmed these findings, with independent prognostic impact of LNI and benefit from adjuvant therapy.

**Conclusions:**

LNI after nCIT is an independent adverse prognostic factor in ESCC. Patients with these high-risk pathological features have markedly worse outcomes but appear to derive significant benefit from adjuvant therapy, supporting its incorporation into postoperative management.

## Graphical Abstract

**Graphical Abstract:**
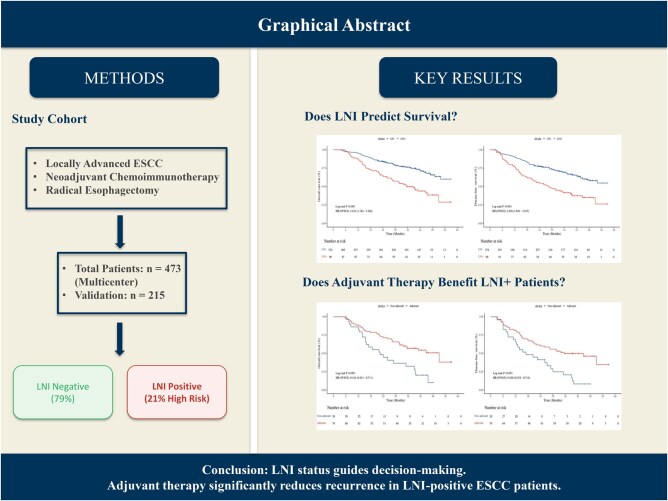

Implications for Practice Lymphovascular and perineural invasion (LNI) are identified as robust independent predictors of poor prognosis in esophageal squamous cell carcinoma after neoadjuvant chemoimmunotherapy. This multicenter study, supported by external validation, reveals that LNI-positive patients exhibit aggressive recurrence patterns but derive significant survival benefit from adjuvant therapy. Consequently, LNI status should serve as a vital pathological biomarker to guide postoperative management. These findings support a paradigm shift toward intensified adjuvant treatment for this high-risk subgroup to improve clinical outcomes.

## Introduction

Esophageal squamous cell carcinoma (ESCC) carries a dismal prognosis, with 5-year survival below 40% for locally advanced disease despite multimodal therapy.^1^^,^^2^ Recently, neoadjuvant chemoimmunotherapy (nCIT) has yielded promising results, achieving higher pathologic complete response (pCR) rates and potentially reducing distant relapse.^3–5^ Nonetheless, a substantial subset of patients treated with nCIT exhibit residual viable tumor and remain at high risk of recurrence.^3^^,^^5^

Robust post-neoadjuvant prognostic markers are therefore needed to guide subsequent therapy. Lymphovascular invasion (LVI)—the presence of tumor cells within lymphatic or vascular channels—and perineural invasion (PNI)—tumor infiltration of the perineural space—are well-recognized adverse pathological features.^6^^,^^7^ Both correlate with advanced stage, nodal metastasis, and poor survival, and their documentation is recommended in the AJCC 8th edition.^8^ A meta-analysis of 27 studies involving 5,740 patients confirmed that lymphatic or vascular invasion predicts significantly poorer overall and recurrence-free survival in ESCC.^6^ Similarly, PNI signifies biologically aggressive disease and is associated with unfavorable outcomes, particularly in patients with residual tumor after neoadjuvant therapy.

However, the prognostic significance of LVI and PNI in the context of neoadjuvant immunotherapy remains uncertain. Immune checkpoint blockade can alter tumor biology, reshape the tumor microenvironment, and modify metastatic dissemination.^9^ A recent study reported a lower incidence of LVI among patients receiving nCIT compared with neoadjuvant chemoradiotherapy (nCRT) (12.5% vs 16.6%), suggesting that immunotherapy may eradicate micrometastatic disease.^10^ Yet, a subset of patients still exhibit LVI and/or PNI on final pathology, identifying a group at particularly high risk of relapse. Evidence on the prognostic value of these features after nCIT—and the potential benefit of adjuvant therapy in this setting—remains limited.

To address this gap, we conducted a multicenter retrospective analysis of 473 patients with ESCC treated with nCIT followed by curative resection. For analysis, the presence of either LVI or PNI was categorized as lymphovascular or neural invasion (LNI). We hypothesized that LNI positivity would predict inferior post-neoadjuvant outcomes and that adjuvant therapy could improve survival in this high-risk subgroup.

## Methods

### Study design and ethics

This multicenter retrospective study included 473 consecutive patients with pathologically confirmed ESCC who underwent neoadjuvant chemoimmunotherapy followed by esophagectomy between January 2021 and June 2023 across 3 institutions: Fujian Medical University Union Hospital, Xiamen First Hospital, and Quanzhou First Hospital. All procedures adhered to the Declaration of Helsinki and were approved by the Ethics Committee of Fujian Medical University Union Hospital (approval no. 2025KY670). The requirement for informed consent was waived due to the retrospective study design.

### Patient selection

Eligibility criteria included (1) newly diagnosed, locally advanced, resectable ESCC (clinical stage T1b–T4a, N0–N+); (2) completion of the planned neoadjuvant chemotherapy combined with a PD-1 inhibitor; and (3) achievement of R0 resection. Exclusion criteria were (1) distant metastasis at initial diagnosis; (2) primary tumor located in the cervical esophagus; (3) prior anticancer therapy before nCIT; (4) perioperative death within 30 days; or (5) incomplete clinicopathologic or follow-up data.

### Staging and treatment protocol

Baseline staging evaluations included endoscopic ultrasound and positron emission tomography/computed tomography (PET/CT). Neoadjuvant therapy typically comprised a platinum–paclitaxel doublet combined with a PD-1 inhibitor (nivolumab, pembrolizumab, sintilimab, camrelizumab, or tislelizumab), administered for 2 to 4 cycles. Transthoracic esophagectomy with systematic lymphadenectomy was performed approximately 4-6 weeks after completing neoadjuvant therapy, provided there was no evidence of disease progression. Postoperative adjuvant therapy—immunotherapy with or without chemotherapy—was recommended at the discretion of a multidisciplinary team, particularly for patients with residual nodal disease or other high-risk pathological features.

### Pathological assessment and definitions

Resected specimens were evaluated by specialized gastrointestinal pathologists. LVI was defined as the presence of tumor cells within an endothelial-lined vascular or lymphatic space on histological examination.^11^^,^^12^ Perineural invasion (PNI) was defined as the presence of tumor cells within the perineural space or nerve sheath.^13^^,^^14^ Tumor regression grade (TRG) was assessed to quantify pathological response to neoadjuvant therapy based on the proportion of viable residual tumor cells (VRTCs): Grade 0, complete response (no VRTCs); Grade 1, near-complete response (<10% VRTCs); Grade 2, partial response (10-50% VRTCs); and Grade 3, minimal or poor response (>50% VRTCs).^14–16^ PD-L1 expression (Combined Positive Score, CPS) was retrospectively collected where available. For analysis, the presence of either LVI or PNI was combined into a single binary variable termed LNI (lymphovascular or neural invasion). Patients were classified as LNI-positive if either feature was present and LNI-negative if neither was detected.

### Outcomes and recurrence patterns

Overall survival (OS) was defined as the interval from surgery to death from any cause. Disease-free survival (DFS) was defined as the interval from surgery to the first recurrence (locoregional or distant) or death, whichever occurred first. Patients without events were censored at the last follow-up. Recurrences were categorized as locoregional (tumor bed, anastomosis, or regional lymph nodes), distant (non-regional lymph nodes or distant organs), or both. Adjuvant therapy referred to any systemic treatment administered postoperatively, including PD-1 inhibitor–based immunotherapy (up to 1 year) with or without chemotherapy.

### Propensity score matching

To minimize selection bias and balance baseline characteristics between LNI-negative and LNI-positive groups, propensity score matching was conducted. Propensity scores for LNI positivity were estimated using logistic regression based on pathological stage, TRG, and receipt of adjuvant therapy. One-to-one nearest-neighbor matching without replacement was applied using a caliper width of 0.2 of the standard deviation of the logit of the propensity score.

### External validation cohort

For external validation, we re-analyzed the publicly available individual-level dataset from Feng et al.^17^ Inclusion criteria matched those of the primary cohort: clinically resectable ESCC, completion of neoadjuvant chemoimmunotherapy (nCIT), R0 resection, and availability of postoperative pathological data including LVI and PNI. Definitions were harmonized where necessary, and LNI positivity was uniformly defined as the presence of either LVI or PNI. Survival analyses were stratified by LNI status.

### Statistical analysis

Continuous variables were expressed as mean ± standard deviation and compared using the Student’s *t*-test. Categorical variables were compared using the χ^2^ test or Fisher’s exact test, as appropriate. Survival curves were generated using the Kaplan–Meier method and compared with the log-rank test. Prognostic factors for OS and DFS were identified using Cox proportional hazards regression. Variables with *P* < .05 in univariate analysis were included in multivariable Cox models. Multicollinearity among variables was assessed using variance inflation factors(VIFs), with a threshold of <2.5 indicating no significant collinearity. Spearman correlation analysis was employed to evaluate biological associations among clinicopathological parameters. Hazard ratios (HRs) and 95% CIs were calculated. Subgroup analyses within the LNI-positive cohort evaluated the effect of adjuvant therapy on survival. Two-sided *P* < .05 was considered statistically significant. Analyses were conducted using R software (version 4.3.1; R Foundation for Statistical Computing).

## Results

### Patient characteristics

Among the 473 patients included, 99 (21.0%) exhibited LVI and/or PNI (LNI+). Specifically, the prevalence was 11.2% for LVI and 16.1% for PNI, while 6.3% of patients exhibited both features concurrently. Baseline demographic and preoperative clinical variables were comparable between groups (Table 1), but pathological responses differed markedly. A pathological complete response (TRG 0) was achieved in 18.2% of LNI− patients vs only 1.0% of LNI+ patients, whereas poor tumor regression (TRG 3) was significantly more frequent in the LNI+ group (50.5% vs 23.8%; *P* < .001). PD-L1 expression (CPS ≥ 1) was observed in 20.3% of the cohort, with a balanced distribution between groups (*P* = .382). The distribution of neoadjuvant immunotherapy agents and treatment cycles was well-balanced between the groups (all *P* > .05; Table S1). LNI+ patients were also more likely to receive adjuvant therapy (70.7% vs 55.4%; *P* = .006). After 1:1 propensity score matching, 89 well-balanced pairs of LNI− and LNI+ patients were obtained. In the matched cohort, adjuvant therapy use was similar (67.4% vs 64.0%; *P* = .636), and all other covariates were evenly distributed.

**Table 1:** Baseline clinicopathologic characteristics of patients with and without lymphovascular or perineural invasion (LNI) before and after propensity score matching.

Variables	Before PSM				After PSM			
	Total (*n* = 473)	LNI- (*n* = 374)	LNI+ (*n* = 99)	*P*	Total (*n* = 178)	LNI- (*n* = 89)	LNI+ (*n* = 89)	*P*
**Age, mean ± SD**	60.27 ± 7.47	60.17 ± 7.43	60.64 ± 7.66	.580	60.77 ± 7.84	60.44 ± 7.95	61.10 ± 7.75	.574
**BMI, mean ± SD**	21.91 ± 3.00	21.95 ± 3.01	21.75 ± 3.01	.574	21.84 ± 3.19	21.98 ± 3.38	21.70 ± 3.00	.566
**Gender, *n* (%)**				.331				.234
** Male**	375 (79.28)	300 (80.21)	75 (75.76)		131 (73.6)	62 (69.66)	69 (77.53)	
** Female**	98 (20.72)	74 (19.79)	24 (24.24)		47 (26.4)	27 (30.34)	20 (22.47)	
**Smoking, *n* (%)**				.260				.652
** No**	201 (42.49)	154 (41.18)	47 (47.47)		81 (45.51)	42 (47.19)	39 (43.82)	
** Yes**	272 (57.51)	220 (58.82)	52 (52.53)		97 (54.49)	47 (52.81)	50 (56.18)	
**Drinking, *n* (%)**				.197				.548
** No**	226 (47.78)	173 (46.26)	53 (53.54)		96 (53.93)	50 (56.18)	46 (51.69)	
** Yes**	247 (52.22)	201 (53.74)	46 (46.46)		82 (46.07)	39 (43.82)	43 (48.31)	
**Hypertension, *n* (%)**				.660				1.000
** No**	426 (90.06)	338 (90.37)	88 (88.89)		156 (87.64)	78 (87.64)	78 (87.64)	
** Yes**	47 (9.94)	36 (9.63)	11 (11.11)		22 (12.36)	11 (12.36)	11 (12.36)	
**Diabetes, *n* (%)**				.990				.213
** No**	449 (94.93)	355 (94.92)	94 (94.95)		172 (96.63)	88 (98.88)	84 (94.38)	
** Yes**	24 (5.07)	19 (5.08)	5 (5.05)		6 (3.37)	1 (1.12)	5 (5.62)	
**ASA, *n* (%)**				.591				.372
** I**	101 (21.35)	79 (21.12)	22 (22.22)		40 (22.47)	23 (25.84)	17 (19.10)	
** II**	299 (63.21)	234 (62.57)	65 (65.66)		113 (63.48)	52 (58.43)	61 (68.54)	
** III**	73 (15.43)	61 (16.31)	12 (12.12)		25 (14.04)	14 (15.73)	11 (12.36)	
**Tumor location, *n* (%)**				.151				.313
** Upper**	78 (16.49)	65 (17.38)	13 (13.13)		23 (12.92)	13 (14.61)	10 (11.24)	
** Middle**	272 (57.51)	219 (58.56)	53 (53.54)		104 (58.43)	55 (61.80)	49 (55.06)	
** Lower**	123 (26)	90 (24.06)	33 (33.33)		51 (28.65)	21 (23.60)	30 (33.71)	
**Tumor size, *n* (%)**				.415				.881
** >5 cm**	237 (50.11)	191 (51.07)	46 (46.46)		85 (47.75)	43 (48.31)	42 (47.19)	
** ≤5 cm**	236 (49.89)	183 (48.93)	53 (53.54)		93 (52.25)	46 (51.69)	47 (52.81)	
**CT stage, *n* (%)**				.307				.541
** 1**	14 (2.96)	13 (3.48)	1 (1.01)		5 (2.81)	4 (4.49)	1 (1.12)	
** 2**	100 (21.14)	83 (22.19)	17 (17.17)		27 (15.17)	12 (13.48)	15 (16.85)	
** 3**	246 (52.01)	188 (50.27)	58 (58.59)		105 (58.99)	51 (57.30)	54 (60.67)	
** 4**	113 (23.89)	90 (24.06)	23 (23.23)		41 (23.03)	22 (24.72)	19 (21.35)	
**CN stage, *n* (%)**				.094				.738
** 0**	86 (18.18)	76 (20.32)	10 (10.10)		17 (9.55)	7 (7.87)	10 (11.24)	
** 1**	132 (27.91)	101 (27.01)	31 (31.31)		59 (33.15)	31 (34.83)	28 (31.46)	
** 2**	186 (39.32)	141 (37.70)	45 (45.45)		83 (46.63)	43 (48.31)	40 (44.94)	
** 3**	69 (14.59)	56 (14.97)	13 (13.13)		19 (10.67)	8 (8.99)	11 (12.36)	
**ypT stage, *n* (%)**				**<.001**				.861
** 0**	65 (13.74)	64 (17.11)	1 (1.01)		4 (2.25)	3 (3.37)	1 (1.12)	
** 1**	81 (17.12)	77 (20.59)	4 (4.04)		8 (4.49)	4 (4.49)	4 (4.49)	
** 2**	89 (18.82)	83 (22.19)	6 (6.06)		10 (5.62)	4 (4.49)	6 (6.74)	
** 3**	222 (46.93)	137 (36.63)	85 (85.86)		151 (84.83)	76 (85.39)	75 (84.27)	
** 4**	16 (3.38)	13 (3.48)	3 (3.03)		5 (2.81)	2 (2.25)	3 (3.37)	
**ypN stage, *n* (%)**				**<.001**				.938
** 0**	237 (50.11)	214 (57.22)	23 (23.23)		48 (26.97)	25 (28.09)	23 (25.84)	
** 1**	134 (28.33)	100 (26.74)	34 (34.34)		70 (39.33)	36 (40.45)	34 (38.20)	
** 2**	78 (16.49)	47 (12.57)	31 (31.31)		43 (24.16)	20 (22.47)	23 (25.84)	
** 3**	24 (5.07)	13 (3.48)	11 (11.11)		17 (9.55)	8 (8.99)	9 (10.11)	
**Differentiation, *n* (%)**				.139				.985
** G1**	145 (30.66)	115 (30.75)	30 (30.30)		55 (30.9)	28 (31.46)	27 (30.34)	
** G2**	227 (47.99)	186 (49.73)	41 (41.41)		77 (43.26)	38 (42.70)	39 (43.82)	
** G3**	101 (21.35)	73 (19.52)	28 (28.28)		46 (25.84)	23 (25.84)	23 (25.84)	
**TRG, *n* (%)**				**<.001**				.653
** TRG 0**	69 (14.59)	68 (18.18)	1 (1.01)		5 (2.81)	4 (4.49)	1 (1.12)	
** TRG 1**	116 (24.52)	99 (26.47)	17 (17.17)		25 (14.04)	12 (13.48)	13 (14.61)	
** TRG 2**	149 (31.5)	118 (31.55)	31 (31.31)		61 (34.27)	30 (33.71)	31 (34.83)	
** TRG 3**	139 (29.39)	89 (23.80)	50 (50.51)		87 (48.88)	43 (48.31)	44 (49.44)	
**Adjuvant, *n* (%)**				**.006**				.636
** No**	196 (41.44)	167 (44.65)	29 (29.29)		61 (34.27)	32 (35.96)	29 (32.58)	
** Yes**	277 (58.56)	207 (55.35)	70 (70.71)		117 (65.73)	57 (64.04)	60 (67.42)	
**PD-L1 CPS, *n* (%)**				.382				.550
** <1**	19 (4.02)	15 (4.01)	4 (4.04)		6 (3.37)	3 (3.37)	3 (3.37)	
** ≥1**	96 (20.30)	71 (18.98)	25 (25.25)		40 (22.47)	17 (19.10)	23 (25.84)	
** Unknown**	358 (75.69)	288 (77.01)	70 (70.71)		132 (74.16)	69 (77.53)	63 (70.79)	

Abbreviations: HR, hazards ratio; TRG, tumor regression grade. Bold values indicate statistical significance.

### Survival outcomes

**Figure 1.**
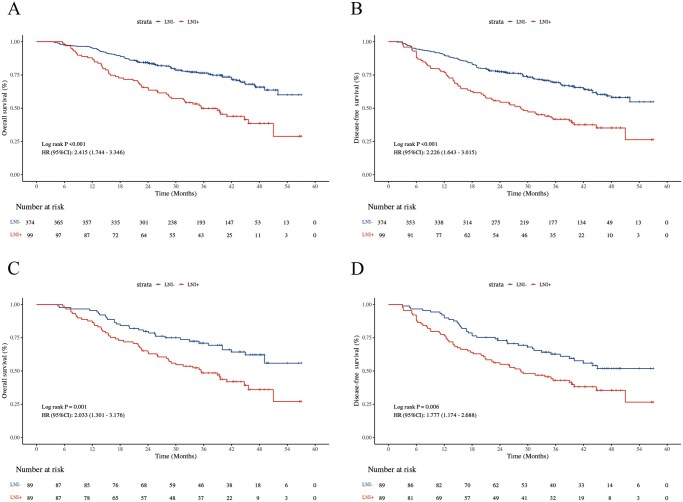
Kaplan–Meier survival curves comparing patients with and without LNI: (A) overall survival (OS) before matching, (B) disease-free survival (DFS) before matching, (C) OS after matching, and (D) DFS after matching.

With a median follow-up of 42 months, survival analyses revealed a consistent trend toward inferior outcomes in patients with microscopic invasive features. As shown in Figure S1, both OS and DFS were significantly poorer in LVI-positive or PNI-positive patients compared to their respective negative counterparts (all log-rank *P* < .001). This prognostic disparity was further consolidated using the integrated LNI status: the 3-year OS rates were 49.8% for LNI+ vs 76.4% for LNI− patients (log-rank *P* < .001), and the 3-year DFS rates were 41.7% vs 69.3%, respectively (log-rank *P* < .001; Figure 1). Notably, most relapses in LNI+ patients occurred within the first postoperative year, underscoring the aggressive biology of LVI/PNI-positive tumors. These findings remained consistent in the matched cohort, where LNI+ patients continued to show significantly inferior OS and DFS compared with LNI− patients (both *P* < .05).

### Cox regression analysis

Univariate Cox regression revealed that LNI positivity (LNI+) was strongly associated with increased risks of both death (HR = 2.42, 95% CI, 1.74-3.35, *P* < .001) and recurrence (HR = 2.23, 95% CI, 1.64-3.01, *P* < .001; Table 2). Additional adverse prognostic factors included pathologic nodal metastasis, higher residual tumor stage, poor regression, and poor differentiation. Univariate analysis showed PD-L1 status was not significantly associated with survival (*P* = .552) and was excluded from multivariable models due to extensive missing data (75.7%).

**Table 2:** Univariate and multivariate Cox regression analyses of overall survival and disease-free survival in the overall cohort.

Variables	OS				DFS			
	Univariate analysis		Multivariate analysis		Univariate analysis		Multivariate analysis	
	HR (95% CI)	*P*	HR (95% CI)	*P*	HR (95% CI)	*P*	HR (95% CI)	*P*
**LNI**								
** LNI-**	1.00 (Reference)		1.00 (Reference)		1.00 (Reference)		1.00 (Reference)	
** LNI+**	2.42 (1.74-3.35)	**<.001**	1.52 (1.06-2.17)	**.022**	2.23 (1.64-3.01)	**<.001**	1.40 (1.01-1.96)	**.045**
**TRG**								
** TRG 0-1**	1.00 (Reference)		1.00 (Reference)		1.00 (Reference)		1.00 (Reference)	
** TRG 2-3**	2.98 (2.01-4.41)	**<.001**	2.05 (1.32-3.17)	**.001**	2.35 (1.68-3.27)	**<.001**	1.65 (1.14-2.38)	**.008**
**ypT stage**								
** T 0-2**	1.00 (Reference)		1.00 (Reference)		1.00 (Reference)		1.00 (Reference)	
** T 3-4**	2.55 (1.82-3.58)	**<.001**	1.29 (0.87-1.91)	.204	2.12 (1.57-2.85)	**<.001**	1.19 (0.84-1.69)	.327
**ypN stage**								
** YpN-**	1.00 (Reference)		1.00 (Reference)		1.00 (Reference)		1.00 (Reference)	
** YpN+**	2.50 (1.79-3.50)	**<.001**	1.73 (1.21-2.47)	**.002**	2.75 (2.02-3.75)	**<.001**	2.08 (1.50-2.89)	**<.001**
**Differentiation**								
** G1**	1.00 (Reference)		1.00 (Reference)		1.00 (Reference)		1.00 (Reference)	
** G2**	0.86 (0.57-1.28)	.447	0.81 (0.54-1.21)	.296	0.83 (0.58-1.17)	.286	0.78 (0.54-1.11)	.160
** G3**	2.49 (1.67-3.71)	**<.001**	2.06 (1.38-3.10)	**<.001**	2.00 (1.39-2.88)	**<.001**	1.64 (1.13-2.38)	**.009**
**Adjuvant**								
** No**	1.00 (Reference)				1.00 (Reference)			
** Yes**	0.81 (0.59-1.11)	.190			0.99 (0.74-1.32)	0.920		
**Age**								
** ≤60**	1.00 (Reference)				1.00 (Reference)			
** >60**	1.05 (0.77-1.44)	.739			0.98 (0.74-1.30)	0.893		
**Gender**								
** Male**	1.00 (Reference)				1.00 (Reference)			
** Female**	0.66 (0.43-1.01)	.056			0.85 (0.59-1.22)	0.377		
**BMI**								
** ＜19**	1.00 (Reference)				1.00 (Reference)			
** 19-25**	0.83 (0.57-1.21)	.334			1.01 (0.70-1.45)	0.961		
** ≥25**	0.83 (0.51-1.37)	.472			1.14 (0.72-1.79)	0.583		
**Smoking**								
** No**	1.00 (Reference)				1.00 (Reference)			
** Yes**	1.24 (0.90-1.71)	.187			1.23 (0.92-1.65)	0.161		
**Drinking**								
** No**	1.00 (Reference)				1.00 (Reference)			
** Yes**	1.34 (0.98-1.84)	.067			1.26 (0.95-1.68)	0.110		
**Hypertension**								
** No**	1.00 (Reference)				1.00 (Reference)			
** Yes**	1.10 (0.66-1.85)	.707			1.22 (0.77-1.92)	0.398		
**Diabetes**								
** No**	1.00 (Reference)				1.00 (Reference)			
** Yes**	0.77 (0.36-1.64)	.496			0.80 (0.41-1.56)	0.516		
**ASA**								
** I**	1.00 (Reference)				1.00 (Reference)			
** II**	1.02 (0.70-1.49)	.928			1.10 (0.78-1.57)	0.582		
** III**	0.93 (0.55-1.58)	.801			0.84 (0.51-1.38)	0.493		
**Tumor location**								
** Upper**	1.00 (Reference)				1.00 (Reference)			
** Middle**	1.37 (0.86-2.19)	.181			1.16 (0.77-1.73)	0.479		
** Lower**	1.38 (0.83-2.32)	.216			1.17 (0.74-1.84)	0.496		

HR, hazards ratio. Multicollinearity was assessed using Variance Inflation Factors, with all values < 1.4, indicating no significant collinearity among variables. Bold values indicate statistical significance.

In the multivariate model adjusting for these covariates, LNI+ remained an independent predictor of poor outcomes for both overall survival (OS; HR = 1.52, 95% CI, 1.06-2.17, *P* = .022) and disease-free survival (DFS; HR = 1.40, 95% CI, 1.01-1.96, *P* = .045). As expected, pathologic nodal metastasis (HR = 1.73, *P* = .002), poor regression (TRG 2-3; HR = 2.05, *P* = .001), and poor differentiation (G3; HR = 2.06, *P* < .001) were strong predictors of inferior outcomes. VIFs (1.03-1.35) confirmed no significant multicollinearity among covariates. Spearman correlation analysis further confirmed that all coefficients were below 0.70 (maximum ρ = 0.51 between ypT and TRG, Figure S2).

In the matched cohort, LNI+ remained significantly associated with elevated risks of death (HR = 1.95, 95% CI, 1.23-3.08, *P* = .005) and recurrence (HR = 1.73, 95% CI, 1.14-2.63, *P* = .011; Table S2). In a stepwise regression (*P* < .05) including LVI, PNI, ypT, ypN, TRG, and differentiation, LVI was retained while ypT and PNI were excluded, indicating LVI’s superior prognostic value over macroscopic depth (Table S3).

### Patterns of recurrence

During follow-up, 113 patients (23.9%) experienced disease recurrence (Table 3). The recurrence rate was markedly higher in the LNI+ group than in the LNI− group (39.4% vs 19.8%; *P* < .001). LNI+ tumors demonstrated a pronounced propensity for locoregional relapse, often accompanied by distant dissemination. Locoregional recurrence—with or without concurrent distant metastases—occurred in 35.4% of LNI+ patients vs 12.6% of LNI− patients. In contrast, the incidence of isolated distant metastasis was comparable between groups (4.0% vs 7.2%). More than half of relapses in the LNI+ cohort involved both local and distant sites, underscoring the aggressive, disseminative phenotype associated with LVI/PNI positivity (*P* < .001 overall).

**Table 3:** Patterns of recurrence in patients with and without LNI in the overall cohort.

Variables	Total (*n* = 473)	LNI- (*n* = 374)	LNI+ (*n* = 99)	*P*
**Supraclavicular, *n* (%)**	15 (3.17)	8 (2.14)	7 (7.07)	**.030**
**Mediastinum, *n* (%)**	44 (9.30)	27 (7.22)	17 (17.17)	**.002**
**Anastomotic, *n* (%)**	21 (4.44)	15 (4.01)	6 (6.06)	.544
**Celiac, *n* (%)**	26 (5.50)	17 (4.55)	9 (9.09)	.078
**Recurrence, *n* (%)**	113 (23.89)	74 (19.79)	39 (39.39)	**<.001**
**Patterns of recurrence, *n* (%)**				**<.001**
** None**	360 (76.11)	300 (80.21)	60 (60.61)	
** DR**	31 (6.55)	27 (7.22)	4 (4.04)	
** LLR**	40 (8.46)	25 (6.68)	15 (15.15)	
** LLR+DR**	42 (8.88)	22 (5.88)	20 (20.20)	

Data are presented as *n* (%). LLR, locoregional recurrence (tumor bed, anastomosis, or regional lymph nodes); DR, distant recurrence (non-regional lymph nodes or distant organs). LLR+DR: Concurrent locoregional and distant recurrence. Bold values indicate statistical significance.

### Adjuvant therapy in LNI+ patients

**Figure 2.**
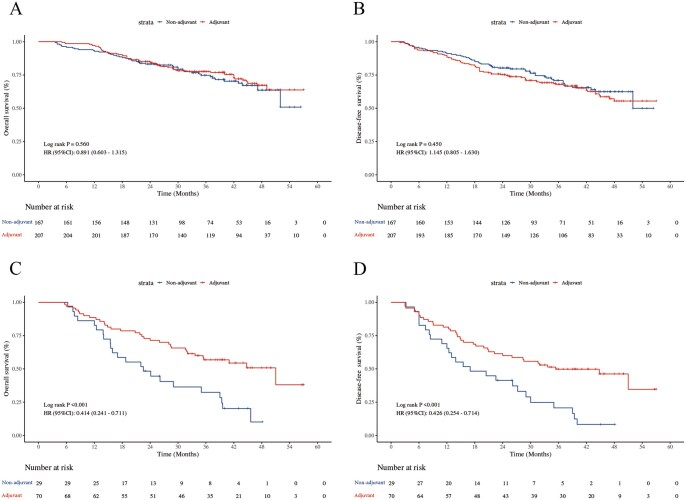
Kaplan–Meier survival curves according to adjuvant therapy: (A) OS in LNI-negative patients, (B) DFS in LNI-negative patients, (C) OS in LNI-positive patients, and (D) DFS in LNI-positive patients.

Among the 99 patients with LNI+, 70 (70.7%) received postoperative adjuvant systemic therapy—predominantly PD-1 inhibitor–based immunotherapy with or without chemotherapy—while 29 (29.3%) did not (Table 4). Baseline characteristics were largely comparable between these subgroups, except for tumor regression grade. Survival analysis demonstrated substantial benefit from adjuvant therapy in LNI+ patients: the 3-year OS was 56.8% with adjuvant therapy vs 32.4% without (*P* = .001, log-rank test), and corresponding 3-year DFS rates were 49.7% vs 20.7% (Figure 2). In multivariate analysis limited to LNI+ patients (Table 5), adjuvant therapy independently conferred a 57% reduction in recurrence or death risk (adjusted HR = 0.43, 95% CI, 0.25-0.74, *P* = .002). By one year post-surgery, nearly half of LNI+ patients without adjuvant therapy had relapsed, whereas most treated patients remained disease-free (Table S4).

**Table 4:** Baseline clinicopathological characteristics of LNI-positive patients according to receipt of adjuvant therapy.

Variables	Total LNI (*n* = 99)	Non-adjuvant (*n* = 29)	Adjuvant (*n* = 70)	*P*
**Age, *n* (%)**				.299
** ≤60**	49 (49.49)	12 (41.38)	37 (52.86)	
** >60**	50 (50.51)	17 (58.62)	33 (47.14)	
**Gender, *n* (%)**				.617
** Male**	75 (75.76)	21 (72.41)	54 (77.14)	
** Female**	24 (24.24)	8 (27.59)	16 (22.86)	
**BMI, *n* (%)**				.570
** ＜19**	23 (23.23)	5 (17.24)	18 (25.71)	
** 19-25**	64 (64.65)	21 (72.41)	43 (61.43)	
** ≥25**	12 (12.12)	3 (10.34)	9 (12.86)	
**Smoking, *n* (%)**				.586
** No**	47 (47.47)	15 (51.72)	32 (45.71)	
** Yes**	52 (52.53)	14 (48.28)	38 (54.29)	
**Drinking, *n* (%)**				.514
** No**	53 (53.54)	17 (58.62)	36 (51.43)	
** Yes**	46 (46.46)	12 (41.38)	34 (48.57)	
**ASA, *n* (%)**				.242
** I**	22 (22.22)	6 (20.69)	16 (22.86)	
** II**	65 (65.66)	17 (58.62)	48 (68.57)	
** III**	12 (12.12)	6 (20.69)	6 (8.57)	
**Tumor location, *n* (%)**				.474
** Upper**	13 (13.13)	2 (6.90)	11 (15.71)	
** Middle**	53 (53.54)	16 (55.17)	37 (52.86)	
** Lower**	33 (33.33)	11 (37.93)	22 (31.43)	
**ypT stage, *n* (%)**				.369
** T 0-2**	11 (11.11)	5 (17.24)	6 (8.57)	
** T 3-4**	88 (88.89)	24 (82.76)	64 (91.43)	
**ypN stage, *n* (%)**				.509
** YpN-**	23 (23.23)	8 (27.59)	15 (21.43)	
** YpN+**	76 (76.77)	21 (72.41)	55 (78.57)	
**Differentiation, *n* (%)**				.198
** G1**	30 (30.30)	11 (37.93)	19 (27.14)	
** G2**	41 (41.41)	8 (27.59)	33 (47.14)	
** G3**	28 (28.28)	10 (34.48)	18 (25.71)	
**TRG, *n* (%)**				.033
** TRG 0-1**	18 (18.18)	9 (31.03)	9 (12.86)	
** TRG 2-3**	81 (81.82)	20 (68.97)	61 (87.14)	
**Pneumonia, *n* (%)**				.564
** No**	59 (59.60)	16 (55.17)	43 (61.43)	
** Yes**	40 (40.40)	13 (44.83)	27 (38.57)	
**Anastomotic leakage, *n* (%)**				.676
** No**	89 (89.90)	25 (86.21)	64 (91.43)	
** Yes**	10 (10.10)	4 (13.79)	6 (8.57)	

**Table 5:** Univariate and multivariate Cox regression analyses of OS and DFS in the LNI-positive cohort.

Variables	OS				DFS			
	Univariate analysis		Multivariate analysis		Univariate analysis		Multivariate analysis	
	HR (95% CI)	*P*	HR (95% CI)	*P*	HR (95% CI)	*P*	HR (95% CI)	*P*
**Adjuvant**								
** No**	1.00 (Reference)		1.00 (Reference)		1.00 (Reference)		1.00 (Reference)	
** Yes**	0.41 (0.24-0.71)	**.001**	0.43 (0.25-0.74)	**.002**	0.43 (0.25-0.71)	**.001**	0.41 (0.24-0.69)	**<.001**
**ypN stage**								
** 0**	1.00 (Reference)		1.00 (Reference)		1.00 (Reference)		1.00 (Reference)	
** 1**	1.65 (0.77-3.54)	.194	1.83 (0.85-3.92)	.122	1.51 (0.72-3.15)	.275	1.69 (0.81-3.55)	.164
** 2**	1.16 (0.52-2.58)	.722	1.32 (0.59-2.95)	.505	1.60 (0.77-3.34)	.212	1.87 (0.89- 3.94)	.099
** 3**	2.67 (1.13-6.30)	**.025**	2.47 (1.04-5.84)	**.040**	2.66 (1.15-6.16)	**.023**	2.68 (1.15-6.22)	**.022**
**ypT stage**								
** T 0-2**	1.00 (Reference)				1.00 (Reference)			
** T 3-4**	1.01 (0.46- 2.24)	0.976			1.24 (0.57-2.74)	.586		
**TRG**								
** TRG 0-1**	1.00 (Reference)				1.00 (Reference)			
** TRG 2-3**	1.02 (0.53-1.97)	.959			1.02 (0.54-1.92)	.943		
**Age**								
** ≤60**	1.00 (Reference)				1.00 (Reference)			
** >60**	1.44 (0.85-2.45)	.175			1.28 (0.78-2.11)	.329		
**Gender**								
** Male**	1.00 (Reference)				1.00 (Reference)			
** Female**	0.51 (0.25-1.04)	.065			0.71 (0.38-1.34)	.292		
**BMI**								
** ＜19**	1.00 (Reference)				1.00 (Reference)			
** 19-25**	0.84 (0.45-1.56)	.585			1.05 (0.57-1.93)	.865		
** ≥25**	0.79 (0.30-2.06)	.631			0.82 (0.32-2.15)	.692		
**Smoking**								
** No**	1.00 (Reference)				1.00 (Reference)			
** Yes**	1.12 (0.66-1.90)	.666			1.04 (0.63-1.72)	.872		
**Drinking**								
** No**	1.00 (Reference)				1.00 (Reference)			
** Yes**	1.27 (0.75-2.15)	.366			1.16 (0.70-1.91)	.564		
**ASA**								
** I**	1.00 (Reference)				1.00 (Reference)			
** II**	1.71 (0.85-3.44)	.132			1.51 (0.79-2.87)	.211		
** III**	2.00 (0.79-5.09)	.145			1.45 (0.59-3.55)	.418		
**Tumor location**								
** Upper**	1.00 (Reference)				1.00 (Reference)			
** Middle**	1.52 (0.63-3.70)	.352			1.63 (0.67-3.92)	.278		
** Lower**	1.82 (0.72-4.58)	.204			2.19 (0.89-5.41)	.089		

Abbreviation: HR, hazards ratio. Bold values indicate statistical significance.

### External validation

To validate these findings, we analyzed an external cohort of 215 patients, including 59 (27.4%) with LNI (Table S5). Consistent results were observed: LNI+ status correlated with significantly worse survival. The 3-year OS rates were 84.0% for LNI− vs 47.5% for LNI+ (*P* < .001), and 3-year DFS rates were 71.8% vs 37.3% (*P* < 0.001; Figure S3A and B). In multivariable Cox analysis adjusting for relevant clinicopathologic covariates, LNI remained an independent prognostic factor for both OS (adjusted HR = 2.38, 95% CI, 1.24-4.56, *P* = .009) and DFS (adjusted HR = 2.15, 95% CI, 1.27-3.67, *P* = .005; Table S6). Among LNI+ patients, adjuvant immunotherapy significantly improved survival (adjusted HR = 0.42 for OS, 95% CI, 0.18-0.96, *P* = .04; adjusted HR = 0.26 for DFS, 95% CI, 0.11-0.61, *P* = .002; Table S7, Figure S4C and D), whereas no survival advantage was seen in LNI− patients (Figure S4A and B).

## Discussion

Our findings demonstrate that LNI after neoadjuvant chemoimmunotherapy is a strong independent predictor of poor prognosis in ESCC. Crucially, our multivariable models, supported by low VIFs (1.03-1.35) and stepwise regression, confirm that LNI provides incremental prognostic value beyond established factors such as ypT stage and TRG. Patients with LNI-positive tumors had approximately double the risk of death or recurrence compared with those without LVI/PNI, even after adjustment for other clinicopathologic factors. LNI positivity was further associated with an aggressive relapse pattern, frequently involving concurrent locoregional and distant failures. Notably, adjuvant therapy significantly improved outcomes in LNI-positive patients, highlighting a potential strategy to mitigate relapse risk in this subgroup.

These results corroborate and extend prior observations of LVI/PNI in esophageal cancer. Zhou et al. reported LVI and PNI rates of 12.5% and 17.8%, respectively, after neoadjuvant chemoradiotherapy, both correlating with shorter OS and DFS.^18^ In their multivariate analysis, LVI independently predicted poor prognosis even in node-negative patients. Consistently, in our nCIT cohort, LNI status remained an independent prognostic factor alongside nodal status. The 21% incidence of LNI in this study parallels the combined LVI/PNI rates (≈20-25%) reported in other series.^19–21^ Notably, the incidence of LVI appears lower following neoadjuvant immunochemotherapy than after chemoradiotherapy. For example, Guo et al. reported a reduced frequency of LVI in patients treated with neoadjuvant immunochemotherapy, and LVI has consistently been linked to unfavorable outcomes.^10^ These findings suggest that immunotherapy may facilitate the eradication of micrometastatic tumor deposits within the lymphovascular system. Nevertheless, a substantial proportion of our patients still exhibited LVI or PNI after nCIT, and their prognosis remained poor in the absence of additional therapy.

The adverse impact of LVI and PNI likely reflects the aggressive tumor biology they represent.^12^^,^^14^ LVI facilitates nodal spread and distant metastasis through vascular invasion, whereas PNI denotes local extension along nerve sheaths and potential neural route dissemination.^11^^,^^13^ Consistent with this, LNI-positive tumors exhibited a marked propensity for multifocal relapse. Interestingly, we observed that distant dissemination in these patients was frequently accompanied by synchronous locoregional failures (LLR+DR), reflecting a systematic failure of both local and systemic control driven by this aggressive phenotype. Given the high rate of locoregional failure (35.4%), postoperative radiotherapy could be a valuable consideration for LNI+ patients, although intensified systemic therapy currently remains our prioritized strategy to address the concurrent high risk of distant metastasis. These findings reinforce that LVI and PNI are histopathologic hallmarks of aggressive disease. In line with prior studies—such as Su et al., who reported a 2.35-fold higher metastatic risk in PNI-positive ESCC after nCRT,^22^ and the meta-analysis by Wang et al. demonstrating inferior survival with lymphatic or vascular invasion^6^—our results emphasize the crucial prognostic value of microscopic vascular and neural invasion in post-neoadjuvant treatment planning.

Findings were externally validated using an independent real-world cohort reported by Feng et al.^17^, which provided individual-level data for re-analysis. The validation cohort reproduced the main results: LNI positivity after neoadjuvant chemoimmunotherapy was consistently associated with inferior OS and DFS, whereas adjuvant systemic therapy significantly improved survival among LNI-positive patients. This external validation supports the robustness and generalizability of our findings across institutions and patient populations, reinforcing LNI as a reliable post-neoadjuvant prognostic biomarker. Furthermore, while emerging molecular and dynamic biomarkers such as PD-L1 expression and ctDNA offer promising insights, their clinical utility is frequently constrained by technical barriers or incomplete data—as evidenced by the 75.7% unknown PD-L1 status in our cohort. In this context, pathological LNI remains an indispensable, cost-effective, and widely accessible morphological indicator that provides critical prognostic value, especially when molecular profiles are unavailable.

A key contribution of this study lies in its therapeutic implications. Adjuvant therapy in LNI-positive patients conferred a 57% reduction in recurrence or death risk, nearly narrowing the survival gap between LNI-positive and LNI-negative patients. Notably, in the neoadjuvant chemoradiotherapy setting, the phase III CheckMate 577 trial established adjuvant nivolumab as a new standard of care by significantly prolonging DFS in esophageal cancer patients with residual disease.^23^ Our findings extend this rationale to the neoadjuvant immunochemotherapy context, suggesting that patients with adverse pathological features (eg, LVI/PNI or nodal metastases) may derive additional benefit from postoperative immunotherapy. This aligns with evolving clinical practice and current guidelines that recommend adjuvant immunotherapy for patients with residual risk despite prior immunotherapy, although direct evidence in the nCIT setting remains forthcoming.^17^ Conversely, while the overlapping survival curves in LNI-negative patients suggest a potential for treatment de-escalation, we do not advocate for the universal omission of therapy. Decisions for LNI-negative individuals must remain personalized through multidisciplinary team discussions, carefully considering other adverse factors such as nodal involvement or tumor regression to avoid potential undertreatment. Further prospective trials are warranted to validate the safety of de-escalation strategies in this morphological low-risk subgroup.

Several limitations should be acknowledged. First, despite rigorous statistical adjustment, the multicenter retrospective design may have introduced heterogeneity in treatment strategies, pathological assessment, and follow-up protocols. Second, the high rate of missing PD-L1 data, as testing was not a mandatory clinical routine, along with the lack of baseline biopsy and ctDNA results, limited the power of our molecular and dynamic sub-analyses. Third, despite rigorous matching, potential selection bias in adjuvant therapy and heterogeneity in postoperative regimens warrant caution in interpretation. Finally, prospective multicenter trials are necessary to validate these findings and optimize individualized postoperative management.

## Conclusion

In summary, LNI after neoadjuvant chemoimmunotherapy represents an independent adverse prognostic factor in ESCC, associated with inferior survival and multifocal recurrence. Adjuvant systemic therapy—particularly PD-1 inhibitor–based immunotherapy—significantly improved outcomes among LNI-positive patients, supporting its integration into postoperative management for this high-risk subgroup. Comprehensive pathological assessment of LVI/PNI should be emphasized to guide individualized treatment strategies, and prospective trials are needed to validate these results and explore complementary therapeutic approaches.

## Supplementary Material

oyag234_Supplementary_Data

## Data Availability

The data underlying this article will be shared on reasonable request to the corresponding author.
