# Supplementary material for: Lymphovascular and perineural invasion guides adjuvant therapy after neoadjuvant chemoimmunotherapy in esophageal squamous cell carcinoma
Source: Oncologist. 2026 Jun 13;31(7):oyag234. doi: 10.1093/oncolo/oyag234 (PMC13331278; doi:10.1093/oncolo/oyag234)
Supplement: oyag234_Supplementary_Data [file oyag234_supplementary_data.zip › 25-Jun-2026_011607_Supplementary_material.docx]

Supplementary Table 1 Summary of neoadjuvant and adjuvant treatment regimens and durations in the overall cohort.

| Variables | Total  (n = 473) | LNI-  (n = 374) | LNI+  (n = 99) | *P* |
| --- | --- | --- | --- | --- |
|  |  |  |  |  |
| Immunotherapy agent, n (%) |  |  |  | 0.278 |
| Nivolumab | 38 (8.03) | 27 (7.22) | 11 (11.11) |  |
| Pembrolizumab | 82 (17.34) | 65 (17.38) | 17 (17.17) |  |
| Camrelizumab | 207 (43.76) | 172 (45.99) | 35 (35.35) |  |
| Tislelizumab | 63 (13.32) | 46 (12.30) | 17 (17.17) |  |
| Sintilimab | 83 (17.55) | 64 (17.11) | 19 (19.19) |  |
| Neoadjuvant treatment cycles,n(%) |  |  |  | 0.207 |
| 2 | 213 (45.03) | 163 (43.58) | 50 (50.51) |  |
| 3 | 218 (46.09) | 180 (48.13) | 38 (38.38) |  |
| 4 | 42 (8.88) | 31 (8.29) | 11 (11.11) |  |
| **Adjuvant therapy** |  |  |  |  |
| POCIT, n(%) | 132 (27.91) | 98 (26.20) | 34 (34.34) | 0.108 |
| POCIT cycles, n(%) |  |  |  | 0.677 |
| 1-2 | 39 (29.55) | 28 (28.57) | 11 (32.35) |  |
| ≥3 | 93 (70.45) | 70 (71.43) | 23 (67.65) |  |
| Adjuvant immune maintenance, n(%) | 145 (30.66) | 109 (29.14) | 36 (36.36) | 0.166 |
| Duration of maintenance, Mean ± SD | 8.65 ± 2.25 | 8.72 ± 2.26 | 8.44 ± 2.22 | 0.532 |

POCIT, Postoperative chemo-immunotherapy.

Supplementary table 2 Univariate and multivariate Cox regression analysis of OS and DFS in the propensity-matched cohort.

| Variables | OS | | | | |  | DFS | | | | |
| --- | --- | --- | --- | --- | --- | --- | --- | --- | --- | --- | --- |
|  |  | Univariate analysis |  | Multivariate analysis |  |  |  | Univariate analysis |  | Multivariate analysis |  |
|  |  | HR (95%CI) | *P* | HR (95%CI) | *P* |  |  | HR (95%CI) | *P* | HR (95%CI) | *P* |
| LNI |  |  |  |  |  |  |  |  |  |  |  |
| LNI- |  | 1.00 (Reference) |  | 1.00 (Reference) |  |  |  | 1.00 (Reference) |  | 1.00 (Reference) |  |
| LNI+ |  | 2.03 (1.30 ~ 3.18) | **0.002** | 1.95 (1.23 ~ 3.08) | **0.005** |  |  | 1.78 (1.17 ~ 2.69) | **0.007** | 1.73 (1.14 ~ 2.63) | **0.011** |
| ypN stage |  |  |  |  |  |  |  |  |  |  |  |
| N 0 |  | 1.00 (Reference) |  | 1.00 (Reference) |  |  |  | 1.00 (Reference) |  | 1.00 (Reference) |  |
| N 1 |  | 1.16 (0.63 ~ 2.11) | 0.638 | 1.27 (0.69 ~ 2.33) | 0.439 |  |  | 1.50 (0.84 ~ 2.68) | 0.168 | 1.59 (0.89 ~ 2.84) | 0.120 |
| N 2 |  | 1.71 (0.92 ~ 3.18) | 0.092 | 1.63 (0.87 ~ 3.05) | 0.131 |  |  | 2.02 (1.10 ~ 3.71) | **0.023** | 1.97 (1.07 ~ 3.63) | **0.030** |
| N 3 |  | 3.14 (1.54 ~ 6.37) | **0.002** | 2.51 (1.23 ~ 5.16) | **0.012** |  |  | 3.24 (1.60 ~ 6.59) | **0.001** | 2.77 (1.36 ~ 5.66) | **0.005** |
| TRG |  |  |  |  |  |  |  |  |  |  |  |
| TRG 0-1 |  | 1.00 (Reference) |  |  |  |  |  | 1.00 (Reference) |  |  |  |
| TRG 2-3 |  | 1.65 (0.85 ~ 3.19) | 0.138 |  |  |  |  | 1.56 (0.85 ~ 2.86) | 0.152 |  |  |
| Adjuvant |  |  |  |  |  |  |  |  |  |  |  |
| No |  | 1.00 (Reference) |  | 1.00 (Reference) |  |  |  | 1.00 (Reference) |  | 1.00 (Reference) |  |
| Yes |  | 0.45 (0.29 ~ 0.70) | **<.001** | 0.43 (0.28 ~ 0.67) | **<.001** |  |  | 0.52 (0.35 ~ 0.79) | **0.002** | 0.50 (0.33 ~ 0.76) | **0.001** |
| Age |  |  |  |  |  |  |  |  |  |  |  |
| ≤60 |  | 1.00 (Reference) |  |  |  |  |  | 1.00 (Reference) |  |  |  |
| ＞60 |  | 1.26 (0.81 ~ 1.95) | 0.304 |  |  |  |  | 1.24 (0.82 ~ 1.87) | 0.305 |  |  |
| Gender |  |  |  |  |  |  |  |  |  |  |  |
| Male |  | 1.00 (Reference) |  |  |  |  |  | 1.00 (Reference) |  |  |  |
| Female |  | 0.58 (0.34 ~ 1.00) | 0.051 |  |  |  |  | 0.89 (0.55 ~ 1.42) | 0.613 |  |  |
| BMI |  |  |  |  |  |  |  |  |  |  |  |
| ＜19 |  | 1.00 (Reference) |  |  |  |  |  | 1.00 (Reference) |  |  |  |
| 19-25 |  | 0.82 (0.50 ~ 1.35) | 0.438 |  |  |  |  | 0.88 (0.54 ~ 1.43) | 0.610 |  |  |
| ≥25 |  | 0.81 (0.41 ~ 1.61) | 0.551 |  |  |  |  | 1.16 (0.62 ~ 2.17) | 0.634 |  |  |
| ASA |  |  |  |  |  |  |  |  |  |  |  |
| I |  | 1.00 (Reference) |  |  |  |  |  | 1.00 (Reference) |  |  |  |
| II |  | 1.47 (0.84 ~ 2.56) | 0.180 |  |  |  |  | 1.25 (0.75 ~ 2.08) | 0.386 |  |  |
| III |  | 1.53 (0.74 ~ 3.19) | 0.254 |  |  |  |  | 1.15 (0.57 ~ 2.30) | 0.704 |  |  |
| Tumour location |  |  |  |  |  |  |  |  |  |  |  |
| Upper |  | 1.00 (Reference) |  |  |  |  |  | 1.00 (Reference) |  |  |  |
| Middle |  | 1.25 (0.61 ~ 2.55) | 0.545 |  |  |  |  | 0.85 (0.46 ~ 1.56) | 0.599 |  |  |
| Lower |  | 1.73 (0.81 ~ 3.67) | 0.155 |  |  |  |  | 1.17 (0.61 ~ 2.24) | 0.644 |  |  |
| ypT stage |  |  |  |  |  |  |  |  |  |  |  |
| T 0-2 |  | 1.00 (Reference) |  |  |  |  |  | 1.00 (Reference) |  |  |  |
| T 3-4 |  | 1.71 (0.79 ~ 3.71) | 0.175 |  |  |  |  | 1.77 (0.86 ~ 3.65) | 0.124 |  |  |
| Differentiation |  |  |  |  |  |  |  |  |  |  |  |
| G1 |  | 1.00 (Reference) |  |  |  |  |  | 1.00 (Reference) |  |  |  |
| G2 |  | 0.88 (0.52 ~ 1.47) | 0.621 |  |  |  |  | 0.96 (0.59 ~ 1.57) | 0.879 |  |  |
| G3 |  | 1.09 (0.63 ~ 1.90) | 0.762 |  |  |  |  | 1.26 (0.74 ~ 2.12) | 0.393 |  |  |
| HR: Hazards Ratio, CI: Confidence Interval | | | | | | | | | | | |

Supplementary Table 3 Multivariable Cox regression analysis using backward stepwise elimination for identifying independent prognostic factors for OS and DFS.

| Variables | OS | | | | |  | DFS | | | | |
| --- | --- | --- | --- | --- | --- | --- | --- | --- | --- | --- | --- |
|  |  | Univariate analysis |  | Multivariate analysis |  |  |  | Univariate analysis |  | Multivariate analysis |  |
|  |  | HR (95% CI) | *P* | HR (95% CI) | *P* |  |  | HR (95% CI) | *P* | HR (95% CI) | *P* |
| LVI |  |  |  |  |  |  |  |  |  |  |  |
| LVI- |  | 1.00 (Reference) |  | 1.00 (Reference) |  |  |  | 1.00 (Reference) |  | 1.00 (Reference) |  |
| LVI+ |  | 2.31 (1.57 ~ 3.41) | **<.001** | 1.54 (1.03 ~ 2.32) | **0.036** |  |  | 2.22 (1.54 ~ 3.21) | **<.001** | 1.49 (1.02 ~ 2.18) | **0.041** |
| PNI |  |  |  |  |  |  |  |  |  |  |  |
| PNI- |  | 1.00 (Reference) |  |  |  |  |  | 1.00 (Reference) |  |  |  |
| PNI+ |  | 2.29 (1.61 ~ 3.26) | **<.001** |  |  |  |  | 2.17 (1.56 ~ 3.02) | **<.001** |  |  |
| TRG |  |  |  |  |  |  |  |  |  |  |  |
| TRG 0-1 |  | 1.00 (Reference) |  | 1.00 (Reference) |  |  |  | 1.00 (Reference) |  | 1.00 (Reference) |  |
| TRG 2-3 |  | 2.98 (2.01 ~ 4.41) | **<.001** | 2.02 (1.31 ~ 3.13) | **0.002** |  |  | 2.35 (1.68 ~ 3.27) | **<.001** | 1.79 (1.27 ~ 2.53) | **<.001** |
| ypT stage |  |  |  |  |  |  |  |  |  |  |  |
| T 0-2 |  | 1.00 (Reference) |  |  |  |  |  | 1.00 (Reference) |  |  |  |
| T 3-4 |  | 2.55 (1.82 ~ 3.58) | **<.001** |  |  |  |  | 2.12 (1.57 ~ 2.85) | **<.001** |  |  |
| ypN stage |  |  |  |  |  |  |  |  |  |  |  |
| YpN- |  | 1.00 (Reference) |  | 1.00 (Reference) |  |  |  | 1.00 (Reference) |  | 1.00 (Reference) |  |
| YpN+ |  | 2.50 (1.79 ~ 3.50) | **<.001** | 1.78 (1.25 ~ 2.53) | **0.001** |  |  | 2.75 (2.02 ~ 3.75) | **<.001** | 2.15 (1.56 ~ 2.98) | **<.001** |
| Differentiation |  |  |  |  |  |  |  |  |  |  |  |
| G1 |  | 1.00 (Reference) |  | 1.00 (Reference) |  |  |  | 1.00 (Reference) |  | 1.00 (Reference) |  |
| G2 |  | 0.86 (0.57 ~ 1.28) | 0.447 | 0.79 (0.53 ~ 1.19) | 0.256 |  |  | 0.83 (0.58 ~ 1.17) | 0.286 | 0.76 (0.54 ~ 1.09) | 0.137 |
| G3 |  | 2.49 (1.67 ~ 3.71) | **<.001** | 2.06 (1.38 ~ 3.09) | **<.001** |  |  | 2.00 (1.39 ~ 2.88) | **<.001** | 1.71 (1.19 ~ 2.47) | **0.004** |
| HR: hazards ratio, CI: confidence interval | | | | | | | | | | | |

Supplementary table 4 Patterns of recurrence in LNI-positive patients according to receipt of adjuvant therapy

| Variables | Total LNI  (n = 99) | Non-adjuvant  (n = 29) | Adjuvant  (n = 70) | P |
| --- | --- | --- | --- | --- |
|  |  |  |  |  |
| Supraclavicular, n(%) | 7 (7.07) | 2 (6.90) | 5 (7.14) | 1.000 |
| Mediastinum, n(%) | 17 (17.17) | 7 (24.14) | 10 (14.29) | 0.373 |
| Anastomotic, n(%) | 6 (6.06) | 3 (10.34) | 3 (4.29) | 0.492 |
| Celiac, n(%) | 9 (9.09) | 3 (10.34) | 6 (8.57) | 1.000 |
| Patterns of recurrence, n(%) |  |  |  | 0.319 |
| None | 60 (60.61) | 14 (48.28) | 46 (65.71) |  |
| DR | 4 (4.04) | 2 (6.90) | 2 (2.86) |  |
| LLR | 15 (15.15) | 6 (20.69) | 9 (12.86) |  |
| LLR+DR | 20 (20.20) | 7 (24.14) | 13 (18.57) |  |

Supplementary Table 5 Baseline characteristics of patients in the external validation cohort.

| Variables | External cohort (n = 215) | LNI-  (n = 156) | LNI+  (n = 59) | *P* |
| --- | --- | --- | --- | --- |
|  |  |  |  |  |
| Age, Mean ± SD | 63.30 ± 6.82 | 63.54 ± 6.83 | 62.64 ± 6.81 | 0.389 |
| BMI, Mean ± SD | 21.68 ± 2.01 | 21.67 ± 1.89 | 21.71 ± 2.32 | 0.900 |
| Gender, n(%) |  |  |  | 0.587 |
| Male | 190 (88.37) | 139 (89.10) | 51 (86.44) |  |
| Female | 25 (11.63) | 17 (10.90) | 8 (13.56) |  |
| Tumour location, n(%) |  |  |  | 0.642 |
| Upper | 20 (9.30) | 14 (8.97) | 6 (10.17) |  |
| Middle | 124 (57.67) | 93 (59.62) | 31 (52.54) |  |
| Lower | 71 (33.02) | 49 (31.41) | 22 (37.29) |  |
| Surgical method, n(%) |  |  |  | 0.918 |
| McKeown | 185 (86.05) | 134 (85.90) | 51 (86.44) |  |
| Ivor-Lewis | 30 (13.95) | 22 (14.10) | 8 (13.56) |  |
| ypT stage n(%) |  |  |  | **<.001** |
| T0 | 65 (30.23) | 62 (39.74) | 3 (5.08) |  |
| T1 | 35 (16.28) | 33 (21.15) | 2 (3.39) |  |
| T2 | 38 (17.67) | 29 (18.59) | 9 (15.25) |  |
| T3 | 57 (26.51) | 26 (16.67) | 31 (52.54) |  |
| T4 | 20 (9.30) | 6 (3.85) | 14 (23.73) |  |
| ypN stage, n(%) |  |  |  | **<.001** |
| N0 | 130 (60.47) | 104 (66.67) | 26 (44.07) |  |
| N1 | 48 (22.33) | 36 (23.08) | 12 (20.34) |  |
| N2 | 26 (12.09) | 10 (6.41) | 16 (27.12) |  |
| N3 | 11 (5.12) | 6 (3.85) | 5 (8.47) |  |
| Differentiation, n(%) |  |  |  | **0.002** |
| G1 | 49 (22.79) | 41 (26.28) | 8 (13.56) |  |
| G2 | 95 (44.19) | 74 (47.44) | 21 (35.59) |  |
| G3 | 71 (33.02) | 41 (26.28) | 30 (50.85) |  |
| ypTNM, n(%) |  |  |  | **<.001** |
| stage 0 | 65 (30.23) | 62 (39.74) | 3 (5.08) |  |
| stage I | 42 (19.53) | 37 (23.72) | 5 (8.47) |  |
| stage II | 20 (9.30) | 5 (3.21) | 15 (25.42) |  |
| stage III | 64 (29.77) | 42 (26.92) | 22 (37.29) |  |
| stage IV | 24 (11.16) | 10 (6.41) | 14 (23.73) |  |
| PCR, n(%) |  |  |  | **<.001** |
| No | 150 (69.77) | 94 (60.26) | 56 (94.92) |  |
| Yes | 65 (30.23) | 62 (39.74) | 3 (5.08) |  |
| Adjuvant, n(%) |  |  |  | **0.006** |
| No | 137 (63.72) | 108 (69.23) | 29 (49.15) |  |
| Yes | 78 (36.28) | 48 (30.77) | 30 (50.85) |  |

Supplementary Table 6 Univariate and multivariable analyses of factors associated with OS and DFS in the external validation cohort.

| Variables | OS | | | | |  | DFS | | | | |
| --- | --- | --- | --- | --- | --- | --- | --- | --- | --- | --- | --- |
|  |  | Univariate analysis |  | Multivariate analysis |  |  |  | Univariate analysis |  | Multivariate analysis |  |
|  |  | HR (95%CI) | *P* | HR (95%CI) | *P* |  |  | HR (95%CI) | *P* | HR (95%CI) | *P* |
| LNI |  |  |  |  |  |  |  |  |  |  |  |
| LNI- |  | 1.00 (Reference) |  | 1.00 (Reference) |  |  |  | 1.00 (Reference) |  | 1.00 (Reference) |  |
| LNI+ |  | 4.25 (2.50 ~ 7.20) | **<.001** | 2.38 (1.24 ~ 4.56) | **0.009** |  |  | 3.38 (2.19 ~ 5.23) | **<.001** | 2.15 (1.27 ~ 3.67) | **0.005** |
| Age |  |  |  |  |  |  |  |  |  |  |  |
| ≤60 |  | 1.00 (Reference) |  |  |  |  |  | 1.00 (Reference) |  |  |  |
| ＞60 |  | 0.65 (0.38 ~ 1.11) | 0.112 |  |  |  |  | 0.69 (0.44 ~ 1.07) | 0.100 |  |  |
| Sex |  |  |  |  |  |  |  |  |  |  |  |
| Female |  | 1.00 (Reference) |  |  |  |  |  | 1.00 (Reference) |  | 1.00 (Reference) |  |
| Male |  | 0.56 (0.28 ~ 1.11) | 0.096 |  |  |  |  | 0.52 (0.30 ~ 0.92) | **0.024** | 0.64 (0.35 ~ 1.17) | 0.145 |
| BMI |  |  |  |  |  |  |  |  |  |  |  |
| ＜19 |  | 1.00 (Reference) |  |  |  |  |  | 1.00 (Reference) |  |  |  |
| 19-25 |  | 0.72 (0.39 ~ 1.33) | 0.293 |  |  |  |  | 0.61 (0.38 ~ 1.00) | 0.050 |  |  |
| ≥25 |  | 0.94 (0.31 ~ 2.85) | 0.910 |  |  |  |  | 0.75 (0.28 ~ 1.97) | 0.558 |  |  |
| Tumour location |  |  |  |  |  |  |  |  |  |  |  |
| Upper |  | 1.00 (Reference) |  |  |  |  |  | 1.00 (Reference) |  | 1.00 (Reference) |  |
| Middle |  | 0.47 (0.21 ~ 1.03) | 0.060 |  |  |  |  | 0.50 (0.26 ~ 0.98) | **0.043** | 0.64 (0.31 ~ 1.32) | 0.223 |
| Lower |  | 0.71 (0.32 ~ 1.60) | 0.408 |  |  |  |  | 0.75 (0.38 ~ 1.49) | 0.408 | 0.58 (0.27 ~ 1.23) | 0.154 |
| Differentiation |  |  |  |  |  |  |  |  |  |  |  |
| G1 |  | 1.00 (Reference) |  | 1.00 (Reference) |  |  |  | 1.00 (Reference) |  | 1.00 (Reference) |  |
| G2 |  | 2.36 (0.97 ~ 5.76) | 0.059 | 1.95 (0.79 ~ 4.84) | 0.149 |  |  | 1.26 (0.67 ~ 2.36) | 0.475 | 1.12 (0.58 ~ 2.18) | 0.734 |
| G3 |  | 3.19 (1.31 ~ 7.77) | **0.011** | 1.76 (0.70 ~ 4.44) | 0.230 |  |  | 2.09 (1.13 ~ 3.88) | **0.019** | 1.41 (0.71 ~ 2.80) | 0.329 |
| Adjuvant |  |  |  |  |  |  |  |  |  |  |  |
| No |  | 1.00 (Reference) |  |  |  |  |  | 1.00 (Reference) |  |  |  |
| Yes |  | 0.78 (0.45 ~ 1.37) | 0.388 |  |  |  |  | 1.00 (0.64 ~ 1.56) | 0.984 |  |  |
| ypT stage |  |  |  |  |  |  |  |  |  |  |  |
| T0 |  | 1.00 (Reference) |  | 1.00 (Reference) |  |  |  | 1.00 (Reference) |  | 1.00 (Reference) |  |
| T1 |  | 4.00 (1.01 ~ 16.01) | 0.050 | 2.29 (0.54 ~ 9.79) | 0.262 |  |  | 1.94 (0.77 ~ 4.90) | 0.159 | 0.95 (0.35 ~ 2.60) | 0.917 |
| T2 |  | 6.44 (1.77 ~ 23.39) | **0.005** | 1.57 (0.36 ~ 6.91) | 0.548 |  |  | 3.38 (1.48 ~ 7.72) | **0.004** | 0.86 (0.31 ~ 2.42) | 0.777 |
| T3 |  | 11.19 (3.37 ~ 37.19) | **<.001** | 2.14 (0.50 ~ 9.11) | 0.302 |  |  | 5.57 (2.66 ~ 11.64) | **<.001** | 1.21 (0.45 ~ 3.26) | 0.708 |
| T4 |  | 22.15 (6.30 ~ 77.83) | **<.001** | 3.27 (0.71 ~ 15.01) | 0.128 |  |  | 11.64 (5.12 ~ 26.48) | **<.001** | 1.85 (0.62 ~ 5.48) | 0.270 |
| ypN stage |  |  |  |  |  |  |  |  |  |  |  |
| N0 |  | 1.00 (Reference) |  | 1.00 (Reference) |  |  |  | 1.00 (Reference) |  | 1.00 (Reference) |  |
| N1 |  | 5.39 (2.63 ~ 11.03) | **<.001** | 4.07 (1.75 ~ 9.46) | **0.001** |  |  | 4.83 (2.77 ~ 8.43) | **<.001** | 4.17 (2.05 ~ 8.48) | **<.001** |
| N2 |  | 13.25 (6.41 ~ 27.40) | **<.001** | 6.50 (2.81 ~ 15.02) | **<.001** |  |  | 10.81 (5.98 ~ 19.55) | **<.001** | 7.00 (3.35 ~ 14.64) | **<.001** |
| N3 |  | 6.44 (2.27 ~ 18.28) | **<.001** | 4.16 (1.30 ~ 13.35) | **0.017** |  |  | 6.13 (2.72 ~ 13.81) | **<.001** | 4.02 (1.52 ~ 10.67) | **0.005** |
| HR: Hazards Ratio, CI: Confidence Interval | | | | | | | | | | | |

Supplementary Table 7 Univariate and multivariable analyses of adjuvant therapy and survival outcomes among LNI-positive patients in the external validation cohort.

| Variables | OS | | | | |  | DFS | | | | |
| --- | --- | --- | --- | --- | --- | --- | --- | --- | --- | --- | --- |
|  |  | Univariate analysis |  | Multivariate analysis |  |  |  | Univariate analysis |  | Multivariate analysis |  |
|  |  | HR (95%CI) | *P* | HR (95%CI) | *P* |  |  | HR (95%CI) | *P* | HR (95%CI) | *P* |
| Adjuvant |  |  |  |  |  |  |  |  |  |  |  |
| No |  | 1.00 (Reference) |  | 1.00 (Reference) |  |  |  | 1.00 (Reference) |  | 1.00 (Reference) |  |
| Yes |  | 0.55 (0.27 ~ 1.11) | 0.096 | 0.42 (0.18 ~ 0.96) | **0.040** |  |  | 0.56 (0.30 ~ 1.07) | 0.080 | 0.26 (0.11 ~ 0.61) | **0.002** |
| ypT stage |  |  |  |  |  |  |  |  |  |  |  |
| T0 |  | 1.00 (Reference) |  |  |  |  |  | 1.00 (Reference) |  | 1.00 (Reference) |  |
| T1 |  | 0.79 (0.07 ~ 8.73) | 0.847 |  |  |  |  | 0.28 (0.03 ~ 2.75) | 0.277 | 0.77 (0.07 ~ 8.64) | 0.835 |
| T2 |  | 0.42 (0.07 ~ 2.55) | 0.348 |  |  |  |  | 0.27 (0.06 ~ 1.21) | 0.087 | 0.08 (0.01 ~ 0.48) | **0.006** |
| T3 |  | 0.64 (0.14 ~ 2.82) | 0.552 |  |  |  |  | 0.37 (0.11 ~ 1.31) | 0.123 | 0.31 (0.07 ~ 1.38) | 0.124 |
| T4 |  | 1.72 (0.38 ~ 7.80) | 0.479 |  |  |  |  | 1.22 (0.35 ~ 4.30) | 0.757 | 0.75 (0.15 ~ 3.71) | 0.722 |
| ypN stage |  |  |  |  |  |  |  |  |  |  |  |
| N0 |  | 1.00 (Reference) |  | 1.00 (Reference) |  |  |  | 1.00 (Reference) |  | 1.00 (Reference) |  |
| N1 |  | 2.13 (0.72 ~ 6.35) | 0.174 | 1.67 (0.49 ~ 5.73) | 0.412 |  |  | 2.34 (0.92 ~ 5.93) | 0.074 | 4.60 (1.50 ~ 14.05) | **0.007** |
| N2 |  | 4.99 (1.98 ~ 12.58) | **<.001** | 4.10 (1.54 ~ 10.92) | **0.005** |  |  | 4.79 (2.12 ~ 10.81) | **<.001** | 5.90 (2.06 ~ 16.88) | **<.001** |
| N3 |  | 8.56 (2.63 ~ 27.89) | **<.001** | 13.57 (3.34 ~ 55.07) | **<.001** |  |  | 4.93 (1.64 ~ 14.81) | **0.004** | 11.62 (3.08 ~ 43.90) | **<.001** |
| Age |  |  |  |  |  |  |  |  |  |  |  |
| ≤60 |  | 1.00 (Reference) |  | 1.00 (Reference) |  |  |  | 1.00 (Reference) |  | 1.00 (Reference) |  |
| ＞60 |  | 0.44 (0.22 ~ 0.90) | **0.024** | 0.52 (0.23 ~ 1.18) | 0.117 |  |  | 0.45 (0.23 ~ 0.85) | **0.014** | 0.58 (0.28 ~ 1.19) | 0.139 |
| Sex |  |  |  |  |  |  |  |  |  |  |  |
| Female |  | 1.00 (Reference) |  |  |  |  |  | 1.00 (Reference) |  |  |  |
| Male |  | 0.52 (0.21 ~ 1.29) | 0.158 |  |  |  |  | 0.55 (0.24 ~ 1.26) | 0.160 |  |  |
| BMI |  |  |  |  |  |  |  |  |  |  |  |
| ＜19 |  | 1.00 (Reference) |  |  |  |  |  | 1.00 (Reference) |  |  |  |
| 19-25 |  | 1.11 (0.47 ~ 2.62) | 0.809 |  |  |  |  | 0.93 (0.44 ~ 1.94) | 0.838 |  |  |
| ≥25 |  | 0.91 (0.24 ~ 3.53) | 0.893 |  |  |  |  | 0.97 (0.30 ~ 3.10) | 0.959 |  |  |
| Tumour location |  |  |  |  |  |  |  |  |  |  |  |
| Upper |  | 1.00 (Reference) |  | 1.00 (Reference) |  |  |  | 1.00 (Reference) |  |  |  |
| Middle |  | 0.41 (0.15 ~ 1.14) | 0.088 | 0.72 (0.20 ~ 2.62) | 0.614 |  |  | 0.52 (0.19 ~ 1.41) | 0.198 |  |  |
| Lower |  | 0.49 (0.17 ~ 1.40) | 0.184 | 0.62 (0.19 ~ 2.03) | 0.427 |  |  | 0.58 (0.21 ~ 1.62) | 0.298 |  |  |
| Differentiation |  |  |  |  |  |  |  |  |  |  |  |
| G1 |  | 1.00 (Reference) |  |  |  |  |  | 1.00 (Reference) |  |  |  |
| G2 |  | 1.04 (0.32 ~ 3.31) | 0.953 |  |  |  |  | 1.17 (0.38 ~ 3.57) | 0.784 |  |  |
| G3 |  | 1.11 (0.37 ~ 3.29) | 0.856 |  |  |  |  | 1.23 (0.42 ~ 3.59) | 0.712 |  |  |
| HR: Hazards Ratio, CI: Confidence Interval | | | | | | | | | | | |
