# Supplementary material for: Lymphovascular and perineural invasion guides adjuvant therapy after neoadjuvant chemoimmunotherapy in esophageal squamous cell carcinoma
Source: Oncologist. 2026 Jun 13;31(7):oyag234. doi: 10.1093/oncolo/oyag234 (PMC13331278; doi:10.1093/oncolo/oyag234)

Table S1 Summary of neoadjuvant and adjuvant treatment regimens and durations in the overall cohort.

| Variables | Total  (n = 473) | LNI-  (n = 374) | LNI+  (n = 99) | *P* |
| --- | --- | --- | --- | --- |
|  |  |  |  |  |
| Immunotherapy agent, n (%) |  |  |  | 0.278 |
| Nivolumab | 38 (8.03) | 27 (7.22) | 11 (11.11) |  |
| Pembrolizumab | 82 (17.34) | 65 (17.38) | 17 (17.17) |  |
| Camrelizumab | 207 (43.76) | 172 (45.99) | 35 (35.35) |  |
| Tislelizumab | 63 (13.32) | 46 (12.30) | 17 (17.17) |  |
| Sintilimab | 83 (17.55) | 64 (17.11) | 19 (19.19) |  |
| Neoadjuvant treatment cycles,n(%) |  |  |  | 0.207 |
| 2 | 213 (45.03) | 163 (43.58) | 50 (50.51) |  |
| 3 | 218 (46.09) | 180 (48.13) | 38 (38.38) |  |
| 4 | 42 (8.88) | 31 (8.29) | 11 (11.11) |  |
| **Adjuvant therapy** |  |  |  |  |
| POCIT, n(%) | 132 (27.91) | 98 (26.20) | 34 (34.34) | 0.108 |
| POCIT cycles, n(%) |  |  |  | 0.677 |
| 1-2 | 39 (29.55) | 28 (28.57) | 11 (32.35) |  |
| ≥3 | 93 (70.45) | 70 (71.43) | 23 (67.65) |  |
| Adjuvant immune maintenance, n(%) | 145 (30.66) | 109 (29.14) | 36 (36.36) | 0.166 |
| Duration of maintenance, Mean ± SD | 8.65 ± 2.25 | 8.72 ± 2.26 | 8.44 ± 2.22 | 0.532 |

POCIT, Postoperative chemo-immunotherapy.

Table S2 Univariate and multivariate Cox regression analysis of OS and DFS in the propensity-matched cohort.

| Variables | OS | | | | |  | DFS | | | | |
| --- | --- | --- | --- | --- | --- | --- | --- | --- | --- | --- | --- |
|  |  | Univariate analysis |  | Multivariate analysis |  |  |  | Univariate analysis |  | Multivariate analysis |  |
|  |  | HR (95%CI) | *P* | HR (95%CI) | *P* |  |  | HR (95%CI) | *P* | HR (95%CI) | *P* |
| LNI |  |  |  |  |  |  |  |  |  |  |  |
| LNI- |  | 1.00 (Reference) |  | 1.00 (Reference) |  |  |  | 1.00 (Reference) |  | 1.00 (Reference) |  |
| LNI+ |  | 2.03 (1.30 ~ 3.18) | **0.002** | 1.95 (1.23 ~ 3.08) | **0.005** |  |  | 1.78 (1.17 ~ 2.69) | **0.007** | 1.73 (1.14 ~ 2.63) | **0.011** |
| ypN stage |  |  |  |  |  |  |  |  |  |  |  |
| N 0 |  | 1.00 (Reference) |  | 1.00 (Reference) |  |  |  | 1.00 (Reference) |  | 1.00 (Reference) |  |
| N 1 |  | 1.16 (0.63 ~ 2.11) | 0.638 | 1.27 (0.69 ~ 2.33) | 0.439 |  |  | 1.50 (0.84 ~ 2.68) | 0.168 | 1.59 (0.89 ~ 2.84) | 0.120 |
| N 2 |  | 1.71 (0.92 ~ 3.18) | 0.092 | 1.63 (0.87 ~ 3.05) | 0.131 |  |  | 2.02 (1.10 ~ 3.71) | **0.023** | 1.97 (1.07 ~ 3.63) | **0.030** |
| N 3 |  | 3.14 (1.54 ~ 6.37) | **0.002** | 2.51 (1.23 ~ 5.16) | **0.012** |  |  | 3.24 (1.60 ~ 6.59) | **0.001** | 2.77 (1.36 ~ 5.66) | **0.005** |
| TRG |  |  |  |  |  |  |  |  |  |  |  |
| TRG 0-1 |  | 1.00 (Reference) |  |  |  |  |  | 1.00 (Reference) |  |  |  |
| TRG 2-3 |  | 1.65 (0.85 ~ 3.19) | 0.138 |  |  |  |  | 1.56 (0.85 ~ 2.86) | 0.152 |  |  |
| Adjuvant |  |  |  |  |  |  |  |  |  |  |  |
| No |  | 1.00 (Reference) |  | 1.00 (Reference) |  |  |  | 1.00 (Reference) |  | 1.00 (Reference) |  |
| Yes |  | 0.45 (0.29 ~ 0.70) | **<.001** | 0.43 (0.28 ~ 0.67) | **<.001** |  |  | 0.52 (0.35 ~ 0.79) | **0.002** | 0.50 (0.33 ~ 0.76) | **0.001** |
| Age |  |  |  |  |  |  |  |  |  |  |  |
| ≤60 |  | 1.00 (Reference) |  |  |  |  |  | 1.00 (Reference) |  |  |  |
| ＞60 |  | 1.26 (0.81 ~ 1.95) | 0.304 |  |  |  |  | 1.24 (0.82 ~ 1.87) | 0.305 |  |  |
| Gender |  |  |  |  |  |  |  |  |  |  |  |
| Male |  | 1.00 (Reference) |  |  |  |  |  | 1.00 (Reference) |  |  |  |
| Female |  | 0.58 (0.34 ~ 1.00) | 0.051 |  |  |  |  | 0.89 (0.55 ~ 1.42) | 0.613 |  |  |
| BMI |  |  |  |  |  |  |  |  |  |  |  |
| ＜19 |  | 1.00 (Reference) |  |  |  |  |  | 1.00 (Reference) |  |  |  |
| 19-25 |  | 0.82 (0.50 ~ 1.35) | 0.438 |  |  |  |  | 0.88 (0.54 ~ 1.43) | 0.610 |  |  |
| ≥25 |  | 0.81 (0.41 ~ 1.61) | 0.551 |  |  |  |  | 1.16 (0.62 ~ 2.17) | 0.634 |  |  |
| ASA |  |  |  |  |  |  |  |  |  |  |  |
| I |  | 1.00 (Reference) |  |  |  |  |  | 1.00 (Reference) |  |  |  |
| II |  | 1.47 (0.84 ~ 2.56) | 0.180 |  |  |  |  | 1.25 (0.75 ~ 2.08) | 0.386 |  |  |
| III |  | 1.53 (0.74 ~ 3.19) | 0.254 |  |  |  |  | 1.15 (0.57 ~ 2.30) | 0.704 |  |  |
| Tumour location |  |  |  |  |  |  |  |  |  |  |  |
| Upper |  | 1.00 (Reference) |  |  |  |  |  | 1.00 (Reference) |  |  |  |
| Middle |  | 1.25 (0.61 ~ 2.55) | 0.545 |  |  |  |  | 0.85 (0.46 ~ 1.56) | 0.599 |  |  |
| Lower |  | 1.73 (0.81 ~ 3.67) | 0.155 |  |  |  |  | 1.17 (0.61 ~ 2.24) | 0.644 |  |  |
| ypT stage |  |  |  |  |  |  |  |  |  |  |  |
| T 0-2 |  | 1.00 (Reference) |  |  |  |  |  | 1.00 (Reference) |  |  |  |
| T 3-4 |  | 1.71 (0.79 ~ 3.71) | 0.175 |  |  |  |  | 1.77 (0.86 ~ 3.65) | 0.124 |  |  |
| Differentiation |  |  |  |  |  |  |  |  |  |  |  |
| G1 |  | 1.00 (Reference) |  |  |  |  |  | 1.00 (Reference) |  |  |  |
| G2 |  | 0.88 (0.52 ~ 1.47) | 0.621 |  |  |  |  | 0.96 (0.59 ~ 1.57) | 0.879 |  |  |
| G3 |  | 1.09 (0.63 ~ 1.90) | 0.762 |  |  |  |  | 1.26 (0.74 ~ 2.12) | 0.393 |  |  |
| HR: Hazards Ratio, CI: Confidence Interval | | | | | | | | | | | |

Table S3 Multivariable Cox regression analysis using backward stepwise elimination for identifying independent prognostic factors for OS and DFS.

| Variables | OS | | | | |  | DFS | | | | |
| --- | --- | --- | --- | --- | --- | --- | --- | --- | --- | --- | --- |
|  |  | Univariate analysis |  | Multivariate analysis |  |  |  | Univariate analysis |  | Multivariate analysis |  |
|  |  | HR (95% CI) | *P* | HR (95% CI) | *P* |  |  | HR (95% CI) | *P* | HR (95% CI) | *P* |
| LVI |  |  |  |  |  |  |  |  |  |  |  |
| LVI- |  | 1.00 (Reference) |  | 1.00 (Reference) |  |  |  | 1.00 (Reference) |  | 1.00 (Reference) |  |
| LVI+ |  | 2.31 (1.57 ~ 3.41) | **<.001** | 1.54 (1.03 ~ 2.32) | **0.036** |  |  | 2.22 (1.54 ~ 3.21) | **<.001** | 1.49 (1.02 ~ 2.18) | **0.041** |
| PNI |  |  |  |  |  |  |  |  |  |  |  |
| PNI- |  | 1.00 (Reference) |  |  |  |  |  | 1.00 (Reference) |  |  |  |
| PNI+ |  | 2.29 (1.61 ~ 3.26) | **<.001** |  |  |  |  | 2.17 (1.56 ~ 3.02) | **<.001** |  |  |
| TRG |  |  |  |  |  |  |  |  |  |  |  |
| TRG 0-1 |  | 1.00 (Reference) |  | 1.00 (Reference) |  |  |  | 1.00 (Reference) |  | 1.00 (Reference) |  |
| TRG 2-3 |  | 2.98 (2.01 ~ 4.41) | **<.001** | 2.02 (1.31 ~ 3.13) | **0.002** |  |  | 2.35 (1.68 ~ 3.27) | **<.001** | 1.79 (1.27 ~ 2.53) | **<.001** |
| ypT stage |  |  |  |  |  |  |  |  |  |  |  |
| T 0-2 |  | 1.00 (Reference) |  |  |  |  |  | 1.00 (Reference) |  |  |  |
| T 3-4 |  | 2.55 (1.82 ~ 3.58) | **<.001** |  |  |  |  | 2.12 (1.57 ~ 2.85) | **<.001** |  |  |
| ypN stage |  |  |  |  |  |  |  |  |  |  |  |
| YpN- |  | 1.00 (Reference) |  | 1.00 (Reference) |  |  |  | 1.00 (Reference) |  | 1.00 (Reference) |  |
| YpN+ |  | 2.50 (1.79 ~ 3.50) | **<.001** | 1.78 (1.25 ~ 2.53) | **0.001** |  |  | 2.75 (2.02 ~ 3.75) | **<.001** | 2.15 (1.56 ~ 2.98) | **<.001** |
| Differentiation |  |  |  |  |  |  |  |  |  |  |  |
| G1 |  | 1.00 (Reference) |  | 1.00 (Reference) |  |  |  | 1.00 (Reference) |  | 1.00 (Reference) |  |
| G2 |  | 0.86 (0.57 ~ 1.28) | 0.447 | 0.79 (0.53 ~ 1.19) | 0.256 |  |  | 0.83 (0.58 ~ 1.17) | 0.286 | 0.76 (0.54 ~ 1.09) | 0.137 |
| G3 |  | 2.49 (1.67 ~ 3.71) | **<.001** | 2.06 (1.38 ~ 3.09) | **<.001** |  |  | 2.00 (1.39 ~ 2.88) | **<.001** | 1.71 (1.19 ~ 2.47) | **0.004** |
| HR: hazards ratio, CI: confidence interval | | | | | | | | | | | |

Table S4 Patterns of recurrence in LNI-positive patients according to receipt of adjuvant therapy

| Variables | Total LNI  (n = 99) | Non-adjuvant  (n = 29) | Adjuvant  (n = 70) | P |
| --- | --- | --- | --- | --- |
|  |  |  |  |  |
| Supraclavicular, n(%) | 7 (7.07) | 2 (6.90) | 5 (7.14) | 1.000 |
| Mediastinum, n(%) | 17 (17.17) | 7 (24.14) | 10 (14.29) | 0.373 |
| Anastomotic, n(%) | 6 (6.06) | 3 (10.34) | 3 (4.29) | 0.492 |
| Celiac, n(%) | 9 (9.09) | 3 (10.34) | 6 (8.57) | 1.000 |
| Patterns of recurrence, n(%) |  |  |  | 0.319 |
| None | 60 (60.61) | 14 (48.28) | 46 (65.71) |  |
| DR | 4 (4.04) | 2 (6.90) | 2 (2.86) |  |
| LLR | 15 (15.15) | 6 (20.69) | 9 (12.86) |  |
| LLR+DR | 20 (20.20) | 7 (24.14) | 13 (18.57) |  |

Table S5 Baseline characteristics of patients in the external validation cohort.

| Variables | External cohort (n = 215) | LNI-  (n = 156) | LNI+  (n = 59) | *P* |
| --- | --- | --- | --- | --- |
|  |  |  |  |  |
| Age, Mean ± SD | 63.30 ± 6.82 | 63.54 ± 6.83 | 62.64 ± 6.81 | 0.389 |
| BMI, Mean ± SD | 21.68 ± 2.01 | 21.67 ± 1.89 | 21.71 ± 2.32 | 0.900 |
| Gender, n(%) |  |  |  | 0.587 |
| Male | 190 (88.37) | 139 (89.10) | 51 (86.44) |  |
| Female | 25 (11.63) | 17 (10.90) | 8 (13.56) |  |
| Tumour location, n(%) |  |  |  | 0.642 |
| Upper | 20 (9.30) | 14 (8.97) | 6 (10.17) |  |
| Middle | 124 (57.67) | 93 (59.62) | 31 (52.54) |  |
| Lower | 71 (33.02) | 49 (31.41) | 22 (37.29) |  |
| Surgical method, n(%) |  |  |  | 0.918 |
| McKeown | 185 (86.05) | 134 (85.90) | 51 (86.44) |  |
| Ivor-Lewis | 30 (13.95) | 22 (14.10) | 8 (13.56) |  |
| ypT stage n(%) |  |  |  | **<.001** |
| T0 | 65 (30.23) | 62 (39.74) | 3 (5.08) |  |
| T1 | 35 (16.28) | 33 (21.15) | 2 (3.39) |  |
| T2 | 38 (17.67) | 29 (18.59) | 9 (15.25) |  |
| T3 | 57 (26.51) | 26 (16.67) | 31 (52.54) |  |
| T4 | 20 (9.30) | 6 (3.85) | 14 (23.73) |  |
| ypN stage, n(%) |  |  |  | **<.001** |
| N0 | 130 (60.47) | 104 (66.67) | 26 (44.07) |  |
| N1 | 48 (22.33) | 36 (23.08) | 12 (20.34) |  |
| N2 | 26 (12.09) | 10 (6.41) | 16 (27.12) |  |
| N3 | 11 (5.12) | 6 (3.85) | 5 (8.47) |  |
| Differentiation, n(%) |  |  |  | **0.002** |
| G1 | 49 (22.79) | 41 (26.28) | 8 (13.56) |  |
| G2 | 95 (44.19) | 74 (47.44) | 21 (35.59) |  |
| G3 | 71 (33.02) | 41 (26.28) | 30 (50.85) |  |
| ypTNM, n(%) |  |  |  | **<.001** |
| stage 0 | 65 (30.23) | 62 (39.74) | 3 (5.08) |  |
| stage I | 42 (19.53) | 37 (23.72) | 5 (8.47) |  |
| stage II | 20 (9.30) | 5 (3.21) | 15 (25.42) |  |
| stage III | 64 (29.77) | 42 (26.92) | 22 (37.29) |  |
| stage IV | 24 (11.16) | 10 (6.41) | 14 (23.73) |  |
| PCR, n(%) |  |  |  | **<.001** |
| No | 150 (69.77) | 94 (60.26) | 56 (94.92) |  |
| Yes | 65 (30.23) | 62 (39.74) | 3 (5.08) |  |
| Adjuvant, n(%) |  |  |  | **0.006** |
| No | 137 (63.72) | 108 (69.23) | 29 (49.15) |  |
| Yes | 78 (36.28) | 48 (30.77) | 30 (50.85) |  |

Table S6 Univariate and multivariable analyses of factors associated with OS and DFS in the external validation cohort.

| Variables | OS | | | | |  | DFS | | | | |
| --- | --- | --- | --- | --- | --- | --- | --- | --- | --- | --- | --- |
|  |  | Univariate analysis |  | Multivariate analysis |  |  |  | Univariate analysis |  | Multivariate analysis |  |
|  |  | HR (95%CI) | *P* | HR (95%CI) | *P* |  |  | HR (95%CI) | *P* | HR (95%CI) | *P* |
| LNI |  |  |  |  |  |  |  |  |  |  |  |
| LNI- |  | 1.00 (Reference) |  | 1.00 (Reference) |  |  |  | 1.00 (Reference) |  | 1.00 (Reference) |  |
| LNI+ |  | 4.25 (2.50 ~ 7.20) | **<.001** | 2.38 (1.24 ~ 4.56) | **0.009** |  |  | 3.38 (2.19 ~ 5.23) | **<.001** | 2.15 (1.27 ~ 3.67) | **0.005** |
| Age |  |  |  |  |  |  |  |  |  |  |  |
| ≤60 |  | 1.00 (Reference) |  |  |  |  |  | 1.00 (Reference) |  |  |  |
| ＞60 |  | 0.65 (0.38 ~ 1.11) | 0.112 |  |  |  |  | 0.69 (0.44 ~ 1.07) | 0.100 |  |  |
| Sex |  |  |  |  |  |  |  |  |  |  |  |
| Female |  | 1.00 (Reference) |  |  |  |  |  | 1.00 (Reference) |  | 1.00 (Reference) |  |
| Male |  | 0.56 (0.28 ~ 1.11) | 0.096 |  |  |  |  | 0.52 (0.30 ~ 0.92) | **0.024** | 0.64 (0.35 ~ 1.17) | 0.145 |
| BMI |  |  |  |  |  |  |  |  |  |  |  |
| ＜19 |  | 1.00 (Reference) |  |  |  |  |  | 1.00 (Reference) |  |  |  |
| 19-25 |  | 0.72 (0.39 ~ 1.33) | 0.293 |  |  |  |  | 0.61 (0.38 ~ 1.00) | 0.050 |  |  |
| ≥25 |  | 0.94 (0.31 ~ 2.85) | 0.910 |  |  |  |  | 0.75 (0.28 ~ 1.97) | 0.558 |  |  |
| Tumour location |  |  |  |  |  |  |  |  |  |  |  |
| Upper |  | 1.00 (Reference) |  |  |  |  |  | 1.00 (Reference) |  | 1.00 (Reference) |  |
| Middle |  | 0.47 (0.21 ~ 1.03) | 0.060 |  |  |  |  | 0.50 (0.26 ~ 0.98) | **0.043** | 0.64 (0.31 ~ 1.32) | 0.223 |
| Lower |  | 0.71 (0.32 ~ 1.60) | 0.408 |  |  |  |  | 0.75 (0.38 ~ 1.49) | 0.408 | 0.58 (0.27 ~ 1.23) | 0.154 |
| Differentiation |  |  |  |  |  |  |  |  |  |  |  |
| G1 |  | 1.00 (Reference) |  | 1.00 (Reference) |  |  |  | 1.00 (Reference) |  | 1.00 (Reference) |  |
| G2 |  | 2.36 (0.97 ~ 5.76) | 0.059 | 1.95 (0.79 ~ 4.84) | 0.149 |  |  | 1.26 (0.67 ~ 2.36) | 0.475 | 1.12 (0.58 ~ 2.18) | 0.734 |
| G3 |  | 3.19 (1.31 ~ 7.77) | **0.011** | 1.76 (0.70 ~ 4.44) | 0.230 |  |  | 2.09 (1.13 ~ 3.88) | **0.019** | 1.41 (0.71 ~ 2.80) | 0.329 |
| Adjuvant |  |  |  |  |  |  |  |  |  |  |  |
| No |  | 1.00 (Reference) |  |  |  |  |  | 1.00 (Reference) |  |  |  |
| Yes |  | 0.78 (0.45 ~ 1.37) | 0.388 |  |  |  |  | 1.00 (0.64 ~ 1.56) | 0.984 |  |  |
| ypT stage |  |  |  |  |  |  |  |  |  |  |  |
| T0 |  | 1.00 (Reference) |  | 1.00 (Reference) |  |  |  | 1.00 (Reference) |  | 1.00 (Reference) |  |
| T1 |  | 4.00 (1.01 ~ 16.01) | 0.050 | 2.29 (0.54 ~ 9.79) | 0.262 |  |  | 1.94 (0.77 ~ 4.90) | 0.159 | 0.95 (0.35 ~ 2.60) | 0.917 |
| T2 |  | 6.44 (1.77 ~ 23.39) | **0.005** | 1.57 (0.36 ~ 6.91) | 0.548 |  |  | 3.38 (1.48 ~ 7.72) | **0.004** | 0.86 (0.31 ~ 2.42) | 0.777 |
| T3 |  | 11.19 (3.37 ~ 37.19) | **<.001** | 2.14 (0.50 ~ 9.11) | 0.302 |  |  | 5.57 (2.66 ~ 11.64) | **<.001** | 1.21 (0.45 ~ 3.26) | 0.708 |
| T4 |  | 22.15 (6.30 ~ 77.83) | **<.001** | 3.27 (0.71 ~ 15.01) | 0.128 |  |  | 11.64 (5.12 ~ 26.48) | **<.001** | 1.85 (0.62 ~ 5.48) | 0.270 |
| ypN stage |  |  |  |  |  |  |  |  |  |  |  |
| N0 |  | 1.00 (Reference) |  | 1.00 (Reference) |  |  |  | 1.00 (Reference) |  | 1.00 (Reference) |  |
| N1 |  | 5.39 (2.63 ~ 11.03) | **<.001** | 4.07 (1.75 ~ 9.46) | **0.001** |  |  | 4.83 (2.77 ~ 8.43) | **<.001** | 4.17 (2.05 ~ 8.48) | **<.001** |
| N2 |  | 13.25 (6.41 ~ 27.40) | **<.001** | 6.50 (2.81 ~ 15.02) | **<.001** |  |  | 10.81 (5.98 ~ 19.55) | **<.001** | 7.00 (3.35 ~ 14.64) | **<.001** |
| N3 |  | 6.44 (2.27 ~ 18.28) | **<.001** | 4.16 (1.30 ~ 13.35) | **0.017** |  |  | 6.13 (2.72 ~ 13.81) | **<.001** | 4.02 (1.52 ~ 10.67) | **0.005** |
| HR: Hazards Ratio, CI: Confidence Interval | | | | | | | | | | | |

Table S7 Univariate and multivariable analyses of adjuvant therapy and survival outcomes among LNI-positive patients in the external validation cohort.

| Variables | OS | | | | |  | DFS | | | | |
| --- | --- | --- | --- | --- | --- | --- | --- | --- | --- | --- | --- |
|  |  | Univariate analysis |  | Multivariate analysis |  |  |  | Univariate analysis |  | Multivariate analysis |  |
|  |  | HR (95%CI) | *P* | HR (95%CI) | *P* |  |  | HR (95%CI) | *P* | HR (95%CI) | *P* |
| Adjuvant |  |  |  |  |  |  |  |  |  |  |  |
| No |  | 1.00 (Reference) |  | 1.00 (Reference) |  |  |  | 1.00 (Reference) |  | 1.00 (Reference) |  |
| Yes |  | 0.55 (0.27 ~ 1.11) | 0.096 | 0.42 (0.18 ~ 0.96) | **0.040** |  |  | 0.56 (0.30 ~ 1.07) | 0.080 | 0.26 (0.11 ~ 0.61) | **0.002** |
| ypT stage |  |  |  |  |  |  |  |  |  |  |  |
| T0 |  | 1.00 (Reference) |  |  |  |  |  | 1.00 (Reference) |  | 1.00 (Reference) |  |
| T1 |  | 0.79 (0.07 ~ 8.73) | 0.847 |  |  |  |  | 0.28 (0.03 ~ 2.75) | 0.277 | 0.77 (0.07 ~ 8.64) | 0.835 |
| T2 |  | 0.42 (0.07 ~ 2.55) | 0.348 |  |  |  |  | 0.27 (0.06 ~ 1.21) | 0.087 | 0.08 (0.01 ~ 0.48) | **0.006** |
| T3 |  | 0.64 (0.14 ~ 2.82) | 0.552 |  |  |  |  | 0.37 (0.11 ~ 1.31) | 0.123 | 0.31 (0.07 ~ 1.38) | 0.124 |
| T4 |  | 1.72 (0.38 ~ 7.80) | 0.479 |  |  |  |  | 1.22 (0.35 ~ 4.30) | 0.757 | 0.75 (0.15 ~ 3.71) | 0.722 |
| ypN stage |  |  |  |  |  |  |  |  |  |  |  |
| N0 |  | 1.00 (Reference) |  | 1.00 (Reference) |  |  |  | 1.00 (Reference) |  | 1.00 (Reference) |  |
| N1 |  | 2.13 (0.72 ~ 6.35) | 0.174 | 1.67 (0.49 ~ 5.73) | 0.412 |  |  | 2.34 (0.92 ~ 5.93) | 0.074 | 4.60 (1.50 ~ 14.05) | **0.007** |
| N2 |  | 4.99 (1.98 ~ 12.58) | **<.001** | 4.10 (1.54 ~ 10.92) | **0.005** |  |  | 4.79 (2.12 ~ 10.81) | **<.001** | 5.90 (2.06 ~ 16.88) | **<.001** |
| N3 |  | 8.56 (2.63 ~ 27.89) | **<.001** | 13.57 (3.34 ~ 55.07) | **<.001** |  |  | 4.93 (1.64 ~ 14.81) | **0.004** | 11.62 (3.08 ~ 43.90) | **<.001** |
| Age |  |  |  |  |  |  |  |  |  |  |  |
| ≤60 |  | 1.00 (Reference) |  | 1.00 (Reference) |  |  |  | 1.00 (Reference) |  | 1.00 (Reference) |  |
| ＞60 |  | 0.44 (0.22 ~ 0.90) | **0.024** | 0.52 (0.23 ~ 1.18) | 0.117 |  |  | 0.45 (0.23 ~ 0.85) | **0.014** | 0.58 (0.28 ~ 1.19) | 0.139 |
| Sex |  |  |  |  |  |  |  |  |  |  |  |
| Female |  | 1.00 (Reference) |  |  |  |  |  | 1.00 (Reference) |  |  |  |
| Male |  | 0.52 (0.21 ~ 1.29) | 0.158 |  |  |  |  | 0.55 (0.24 ~ 1.26) | 0.160 |  |  |
| BMI |  |  |  |  |  |  |  |  |  |  |  |
| ＜19 |  | 1.00 (Reference) |  |  |  |  |  | 1.00 (Reference) |  |  |  |
| 19-25 |  | 1.11 (0.47 ~ 2.62) | 0.809 |  |  |  |  | 0.93 (0.44 ~ 1.94) | 0.838 |  |  |
| ≥25 |  | 0.91 (0.24 ~ 3.53) | 0.893 |  |  |  |  | 0.97 (0.30 ~ 3.10) | 0.959 |  |  |
| Tumour location |  |  |  |  |  |  |  |  |  |  |  |
| Upper |  | 1.00 (Reference) |  | 1.00 (Reference) |  |  |  | 1.00 (Reference) |  |  |  |
| Middle |  | 0.41 (0.15 ~ 1.14) | 0.088 | 0.72 (0.20 ~ 2.62) | 0.614 |  |  | 0.52 (0.19 ~ 1.41) | 0.198 |  |  |
| Lower |  | 0.49 (0.17 ~ 1.40) | 0.184 | 0.62 (0.19 ~ 2.03) | 0.427 |  |  | 0.58 (0.21 ~ 1.62) | 0.298 |  |  |
| Differentiation |  |  |  |  |  |  |  |  |  |  |  |
| G1 |  | 1.00 (Reference) |  |  |  |  |  | 1.00 (Reference) |  |  |  |
| G2 |  | 1.04 (0.32 ~ 3.31) | 0.953 |  |  |  |  | 1.17 (0.38 ~ 3.57) | 0.784 |  |  |
| G3 |  | 1.11 (0.37 ~ 3.29) | 0.856 |  |  |  |  | 1.23 (0.42 ~ 3.59) | 0.712 |  |  |
| HR: Hazards Ratio, CI: Confidence Interval | | | | | | | | | | | |

Figure S1 Kaplan–Meier survival curves stratified by different invasive pathological features. (A) Overall survival and (B) disease-free survival stratified by lymphovascular invasion (LVI) status. (C) Overall survival and (D) disease-free survival stratified by perineural invasion (PNI) status. (E) Overall survival and (F) disease-free survival stratified by LNI-negative, LVI or PNI-positive (single positive), and LVI and PNI-positive (double positive) status.


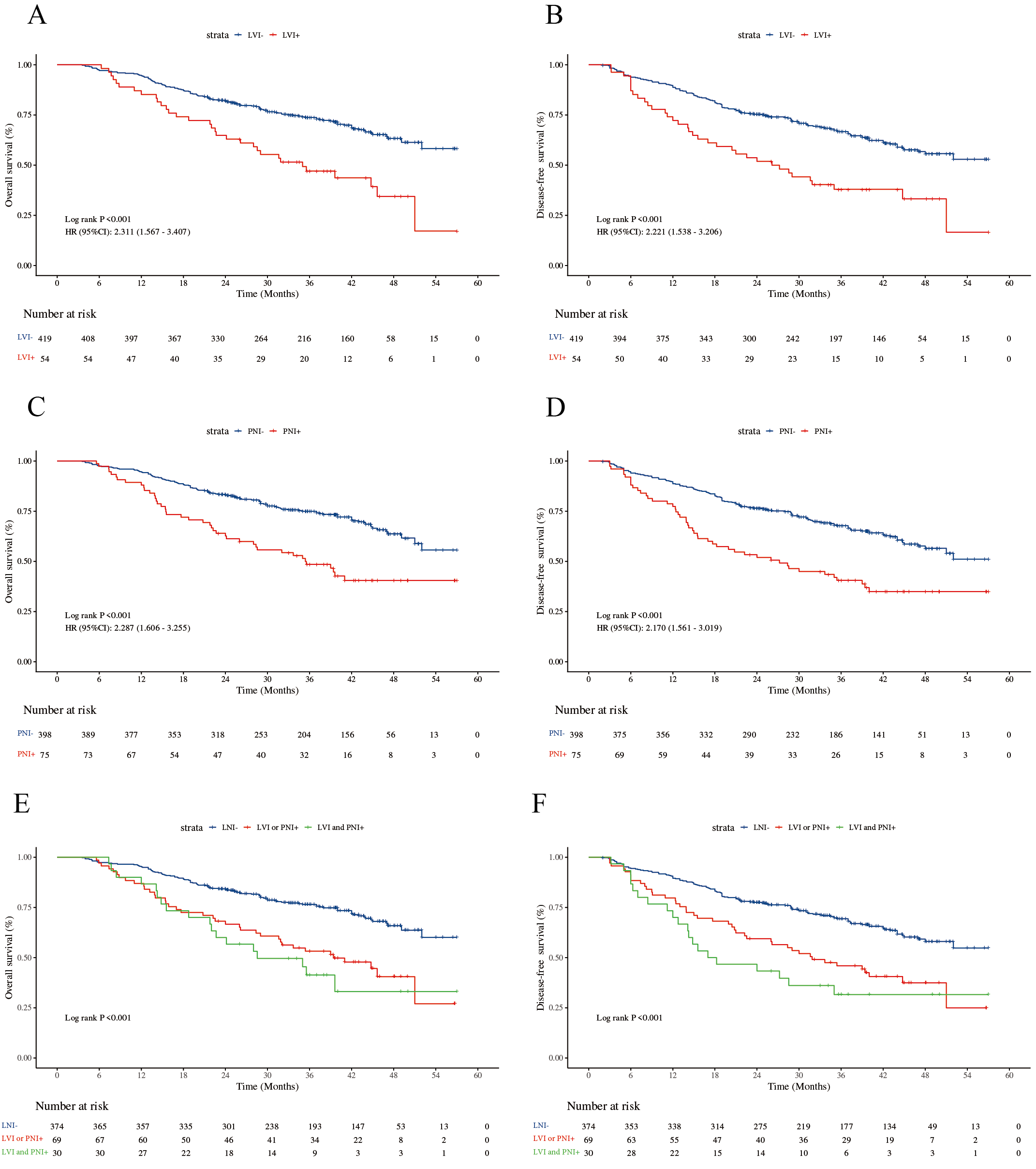


Figure S2 Spearman correlation heatmap of clinicopathological prognostic factors. The heatmap displays the Spearman correlation coefficients (Rho) between key pathological features, including lymphovascular or neural invasion (LNI), pathological T stage (ypT), pathological N stage (ypN), tumor regression grade (TRG), and histological differentiation (G).


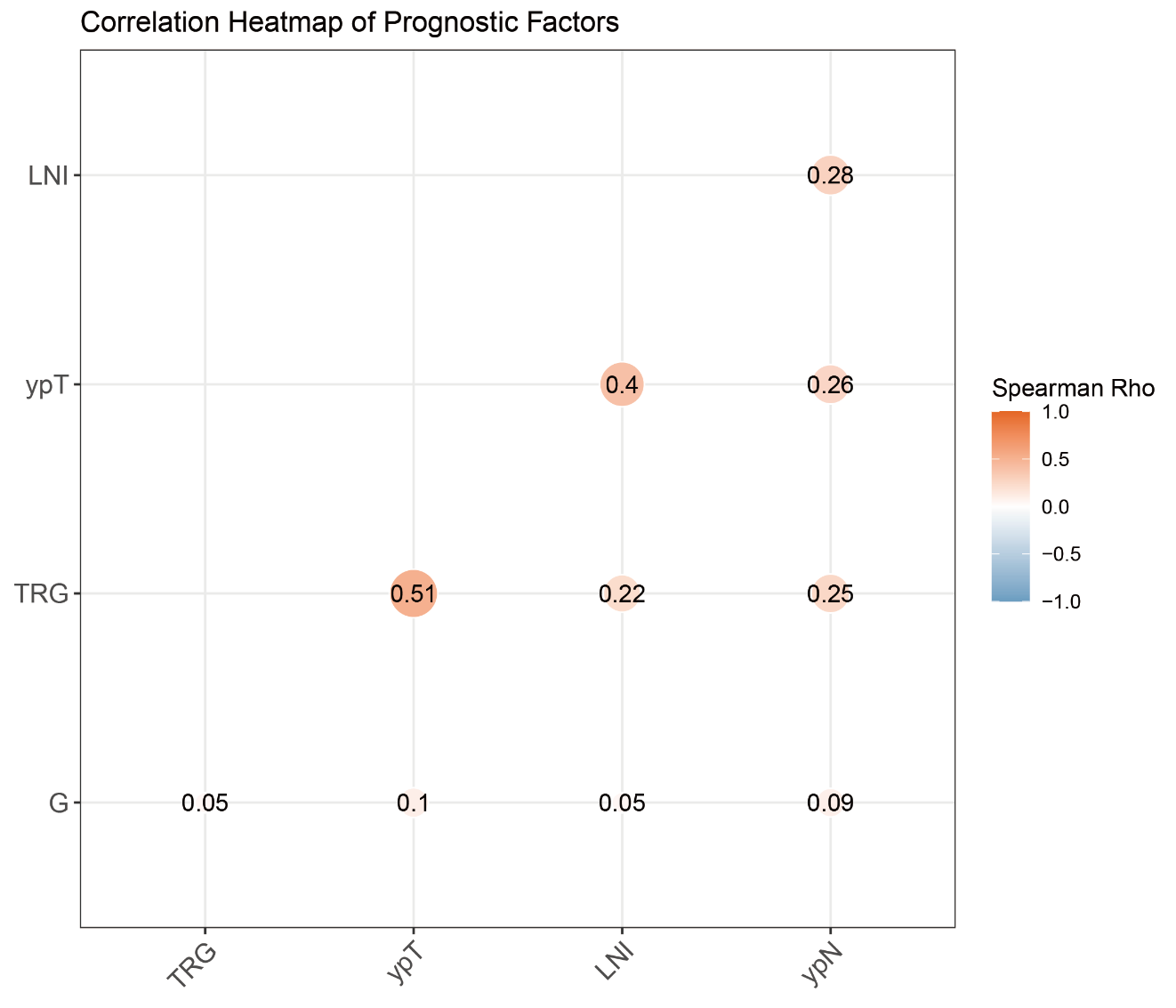


Figure S3 Overall survival (A) and disease-free survival (B) curves according to LNI status in the external validation cohort.


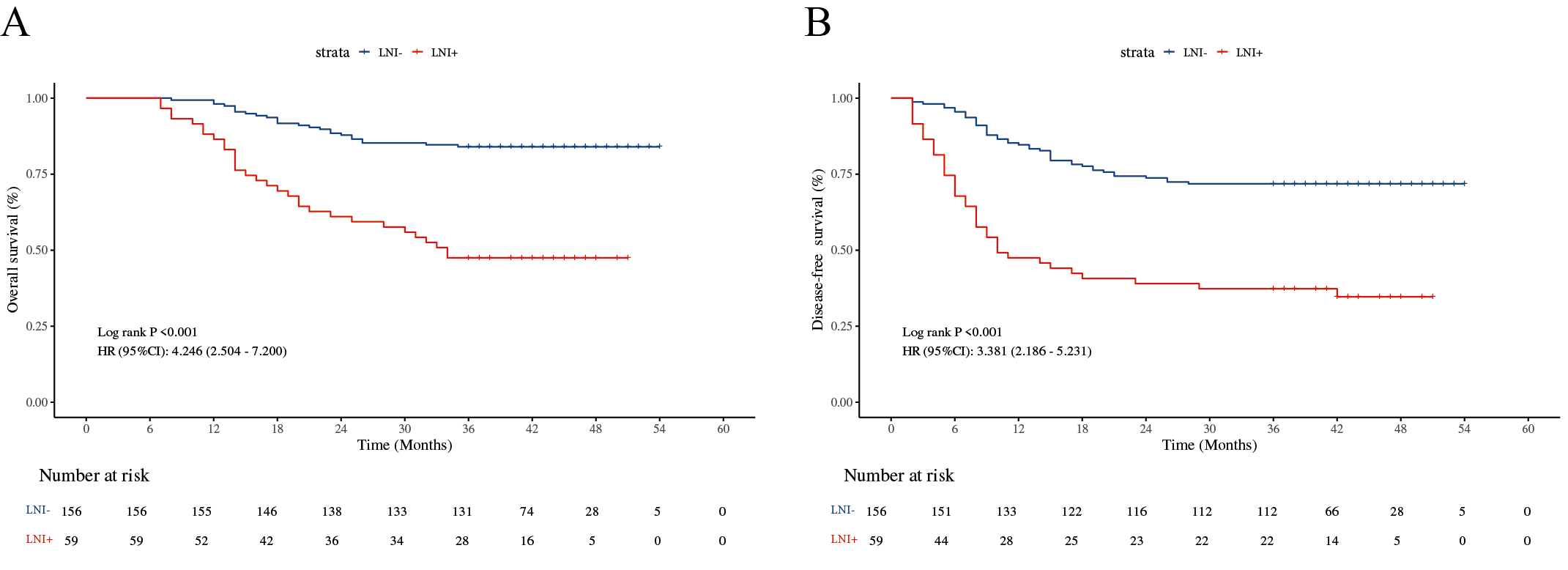


Figure S4 Survival outcomes according to adjuvant therapy stratified by LNI status in the external validation cohort. (A–B) Overall and disease-free survival among LNI-negative patients; (C–D) overall and disease-free survival among LNI-positive patients.


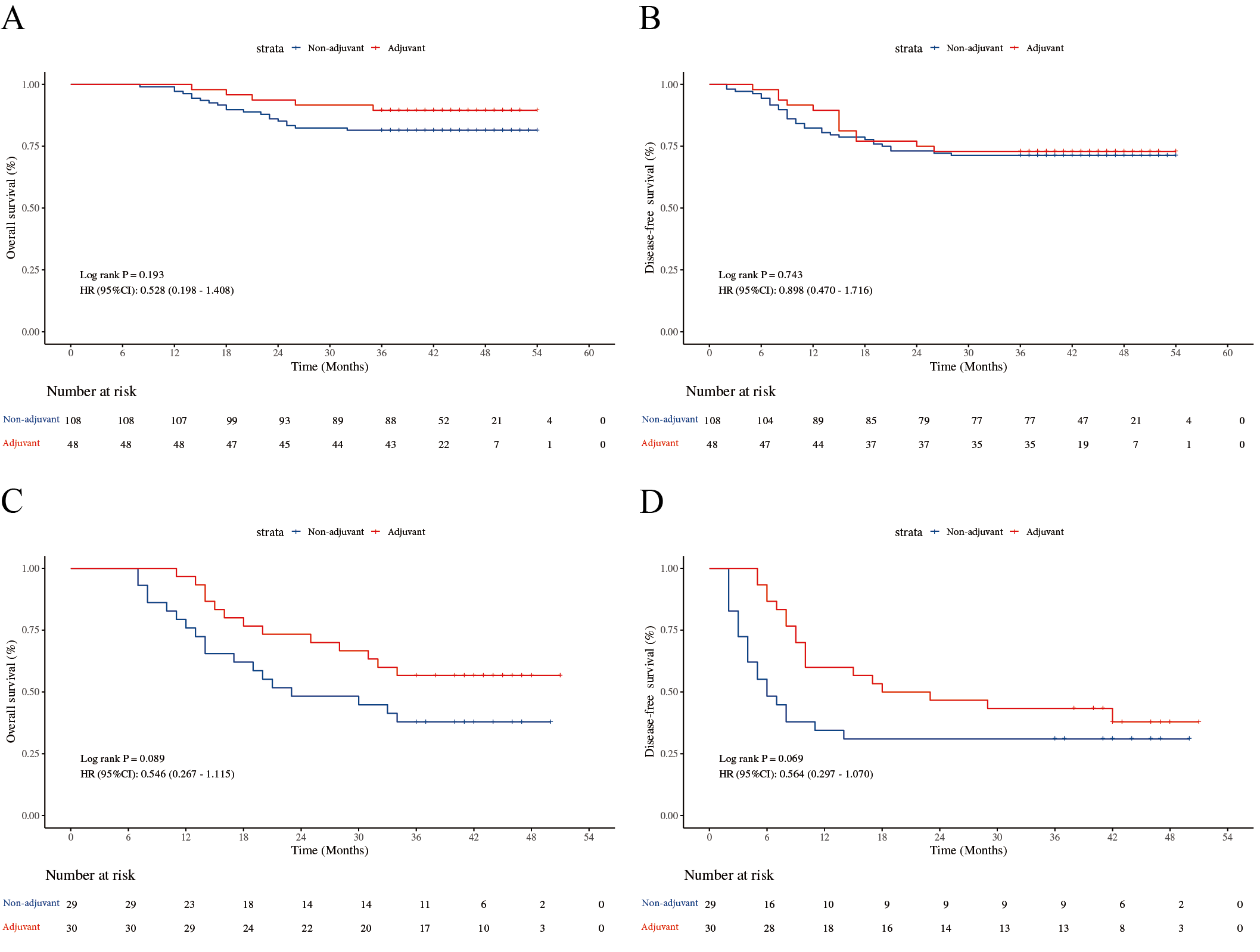

Supplement: oyag234_Supplementary_Data [file oyag234_supplementary_data.zip › Supplementary_material.docx]
